# Emergence of Ebola virus disease in a french acute care setting: a simulation study based on documented inter-individual contacts

**DOI:** 10.1038/srep36301

**Published:** 2016-11-09

**Authors:** Philippe Vanhems, Rosette Von Raesfeldt, René Ecochard, Nicolas Voirin

**Affiliations:** 1 Service d’Hygiène, Epidémiologie et Prévention, Hôpital Edouard Herriot, Hospices Civils de Lyon, F-69437 Lyon, France; 2 Laboratoire des Pathogènes Emergents – Fondation Mérieux, Centre International de Recherche en Infectiologie, Institut National de la Santé et de la Recherche Médicale U1111, Centre National de la Recherche Scientifique, UMR5308, Ecole Normale Supérieure de Lyon, Université Claude Bernard Lyon 1, 21, Avenue Tony Garnier, 69007 Lyon, France; 3 Université de Lyon, F-69000 Lyon, France; 4 Université Lyon 1, F-69100 Villeurbanne, France; 5 Service de Biostatistique, Hospices Civils de Lyon, F-69003 Lyon, France; 6 Laboratoire de Biométrie et Biologie Evolutive, Equipe Biostatistique-Santé, Centre National de la Recherche Scientifique, UMR5558, F-69100 Villeurbanne, France

## Abstract

The potential spread of nosocomial Ebola virus disease (EVD) in non-outbreak areas is not known. The objective was to use detailed contact data on patients and healthcare workers (HCW) to estimate emergence probability and secondary incident cases (SIC) of EVD after hospitalization of an index case with undetected EVD. Contact data were collected through RFID devices used by patients and HCW during hospital care. A “susceptible-exposed-infected” model was used. Emergence probability, ranged from 7% to 84%. A plateau around 84% was observed. Emergence probability was proportional to time exposed to the dry phase of patients with nonspecific symptoms. Nurses were at higher risk of nosocomial EVD than physicians with around 60% emergence probability in this subgroup. The risk of nosocomial EVD in non-outbreak areas might be substantial if no preventive measures are implemented when asymptomatic patients or those with mild symptoms are hospitalized.

Hospital-acquired Ebola virus disease (EVD) has been reported previously^1^,^2^ and during the last outbreak in West Africa^3^,^4^. Contact between EVD patients and healthcare workers (HCW) contributes to its hospital spread^5^, but the dynamics of EVD transmission in healthcare remains complex.

In outbreak areas, symptomatic patients are sources of infection among HCW^1^,^2^, and transmission may be facilitated during the dry phase of the infection, when patients do not exhibit specific signs of EVD^6^,^7^. EVD transmission during hospitalization occurs because of under-diagnosis, failure of differential diagnosis^8^,^9^, repeated exposure to body fluids^10^, especially in intensive care units, during laboratory testing procedures, and inappropriate or delayed infection control measures^2^.

In non-outbreak areas, defined as hospitals in the world outside of an official outbreak zone, nosocomial cases of EVD might arise when a patient or an infected HCW is transferred to a care center^8^ or if a patient presents at hospital with nonspecific EVD symptoms. Few epidemiological data are available to estimate the potential emergence of EVD in medical units located in non-outbreak areas. In addition, the dynamics of spreading infections in hospitals, including EVD, may differ according to contact patterns between HCW and patients. During outbreaks, observational data on contacts can be collected to describe EVD spread in detail outside and inside hospitals^3^,^4^, but similar data are lacking in non-outbreak areas. Mathematical modeling with network contact data could provide epidemiological information on potential EVD spread during hospitalization^11^,^12^.

We used detailed contact data on patients and HCW to estimate the emergence probability of EVD and the number of secondary cases after admission of an index patient with undetected EVD.

## Methods

### Study population

Our analysis relied on contact data collected on 29 patients and 46 HCW, including 27 nurses and 11 physicians at Hôpital Edouard Herriot in Lyon, France^12^. During 5 consecutive days, including nights, each patient wore an electronic sensor (radio frequency identification) which recorded face-to-face interactions with all other persons in the ward. With this technology, individuals wear the devices on their chest so that signals exchange between devices is only possible in a radius of 1–1.5 meters and when they are facing each other, as the human body acts as a shield at the radio frequency used. The system detects and records close-range encounters during which a communicable disease infection could be transmitted, for example, by contact^12^. The protocol was approved by the French national bodies responsible for ethics and privacy, the “Commission Nationale de l’Informatique et des Libertés” (CNIL, http://www.cnil.fr) and the “Comité de Protection des personnes” ( http://www.cppsudest2.com/) of our hospital. A written informed consent was obtained from all subjects. The methods were carried out in accordance with the relevant guidelines and the french law (loi du 9 août 2004).

### Mathematical modelling

**Figure 1:**
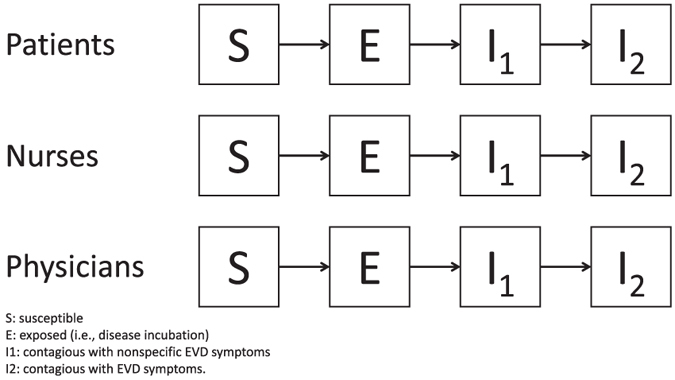
Schematic diagram of the “susceptible -infected” model of EVD emergence in a single hospital ward. The index patient may transmit the disease to S individuals while in I_1_ but as soon as he/she enters the I_2_ stage, he/she is assumed to be 100% detected and isolated with no delay. In other words, once the index case enters the I_2_ compartment, we consider that he/she do not participate in transmission anymore because of 100% effective control measures implemented.

EVD emergence was studied in a mathematical model (Fig. 1) that considered 3 populations interacting in the hospital ward. All patients, nurses and physicians were potentially susceptible to infection, infected with EVD and incubating (E), contagious with nonspecific EVD symptoms (I_1_) or contagious with EVD symptoms (I_2_). In this model, we considered that patients could transmit the disease to susceptible individuals while in I_1_, but as soon as they entered the I_2_ stage, they were assumed to be 100% detected, and isolated without delay. In other words, once individuals entered the I_2_ disease compartment, we supposed that they did not participate in transmission anymore because of 100% effective control measures implemented.

Bed occupancy was presumed to be 100%, and the HCW population was constant. Each population was considered to be homogeneous, i.e., we ignored behavioural variations between individuals. It was equally probable for each individual to be in contact with others, according to observed contact matrices derived from contact data collected by electronic sensors^12^. Patient and HCW turnover was ignored since we assumed that it was negligible in comparison to the short timeframe of simulations.

### Model parameters

Model parameters are presented in Table 1. 29 patients, 27 nurses and 11 physicians interacted with each other, according to observed contact matrices W_*ij*_ (Table 2) and 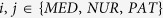.

We studied the impact of varying 3 parameters. To assess the outcome of prevention and control measures in interrupting transmission, we varied transmission probability per contact (*p*) between 0.001 and 1. To investigate how reduced contact intensity between nurses and patients influences the results, we modified the daily number of contacts from 27.3 (average value observed) to 5 (minimum plausible value) per day as preventive measures. To assess how the duration of the dry phase with nonspecific EVD symptoms (I_1_ compartment) affects EVD emergence, we altered I_1_ duration from 1 to 3 days. We also conducted a sensitivity analysis for the duration of the incubation period.

As infection emergence largely depends on chance effects among small populations, as in hospital wards, a stochastic approach has been shown to be essential^11^. In stochastic models, while the underlying epidemiological paradigm remains the same, the outcome will be different in each simulation because the timing of events and the events that occur are chance processes. Model transitions and their rates are reported in Table 3. We relied on the Gillespie algorithm to study a wide range of possible emergence and outbreaks^13^. We ran 2,000 simulations, and for each simulation, we introduced 1 index case patient in the I_1_ stage. Simulation were undertaken with the ssa() function of the GillespieSSA package^14^ using R software ( http://www.R-project.org/).

### Model outputs

A secondary case was defined as a patient, nurse or physician entering in the I_1_ compartment. We calculated EVD emergence probability as the number of simulations with at least 1 secondary incident case (SIC), among patients, nurses and physicians, divided by the total number of simulations. We also calculated EVD emergence probability according to the population where it emerged (patients, nurses or physicians). To assess variability of the emergence probability, we estimated the distribution of the emergence probability using non parametric bootstrap and reported mean and min-max band of the emergence probability. When emergence occurred, we summarized the number of SIC, using mean, minimum and maximum across simulations.

## Results

### Emergence probability and number of SIC according to transmission probability

**Figure 2:**
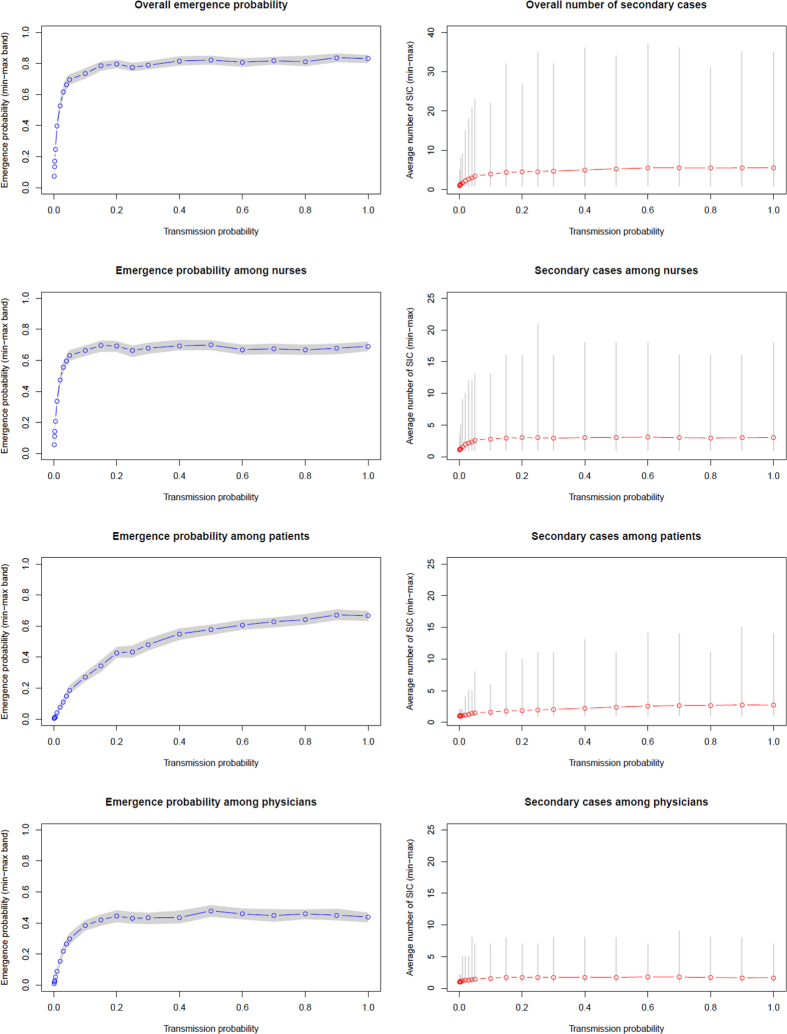
Overall emergence probability and overall number of SIC in a single hospital ward according to EVD transmission probability.

Overall EVD emergence probability in hospital ward ranged from 7% to 84% with an average number of SIC from varying from 1 (min 1 - max 3) to 6^1–37^, with transmission probability rising from 0.001 to 1 (Fig. 2). Emergence probability increased rapidly with low transmission probability and reached a plateau at around 84%, with low variability. The average number of SIC was multiplied by 6 with high transmission compared to low transmission, but more importantly, maximum number of SIC increased from 3 to 37, indicating the potential for large outbreaks. Analysis of emergence by population (patients, nurses, and physicians) is reported in Fig. 2 and numerically in Supplementary Information. Emergence probability (Fig. 1) and average SIC (Supplementary Information) increased significantly among PAT mainly in case of high transmission to reach about 65% and 3 SIC respectively.

### Emergence probability and number of SIC according to the number of contacts that nurses had with undiagnosed infected patients

**Figure 3:**
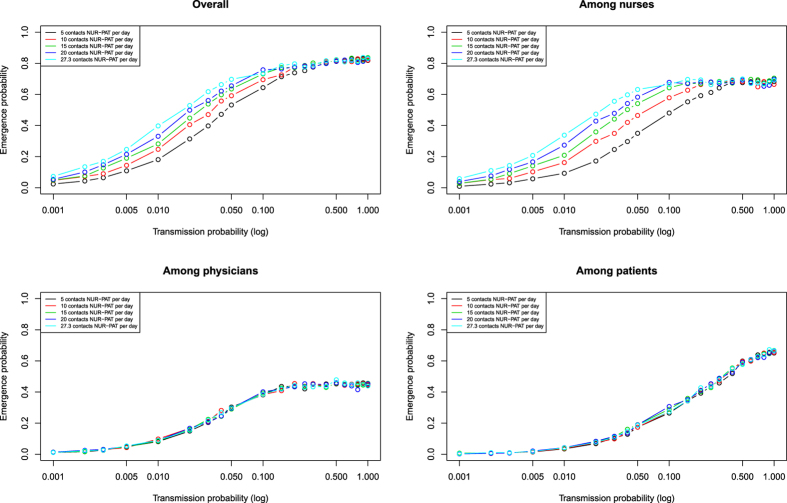
Emergence probability of EVD in a single hospital ward according to transmission probability and number of nurse (NUR) contacts with patients (PAT) by population.

Emergence probability decreased as daily contacts nurses had with patients diminished from 27.3 to 5 (Fig. 3). Low variability was observed in this result (Supplementary Information). Similarly, the average number of SIC declined as daily contacts nurses had with patients decreased (Supplementary Information). Emergence probability and average SIC number among NUR reached maxima of about 60% (Fig. 3) and 2 SIC (Supplementary Information) in case of high transmission and independently of contact numbers of NUR with PAT.

### Emergence probability and number of SIC according to the duration of the dry phase of EVD of the index case

**Figure 4:**
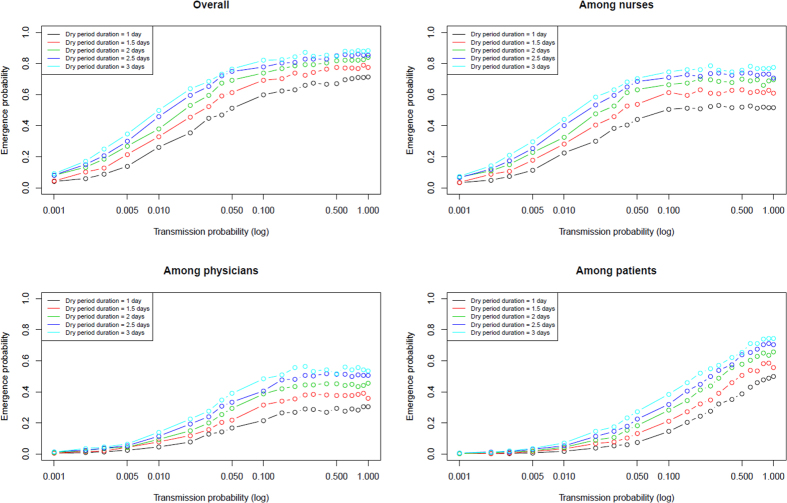
Emergence probability of EVD in a single hospital ward according to transmission probability and duration of the dry phase of the index case.

Emergence probability was proportional to the duration of the dry phase of EVD of the index case, and this effect persisted in case of high transmission (Fig. 4). Again, low variability was observed in this result (Supplementary Information). Similarly, the average number of SIC declined as duration of the dry phase of EVD of the index case decreased (Supplementary Information).

## Discussion

Our objective was to estimate the potential of EVD emergence in an acute care hospital ward located in non-outbreak settings after admission of a undetected patient. The modelling scenario was the under-diagnosis or misdiagnosis of EVD associated with the dry phase of EVD disease at admission because of limited experiences of HCW facing such patients. These results are in line with findings in outbreak areas where EVD among HCW has been documented^3^, which reported that, in areas without community EVD outbreaks, nurses are exposed the most to nosocomial EVD. Control measures, such as contact reduction, decreased transmission probability and early detection, may be key to rapidly avoiding the spread of EVD in hospitals.

Transmission probability can be lowered by prevention measures, such as wearing masks and gloves, hand hygiene, and environmental disinfection. The improvement of care organization in the units in which patient were hospitalized contribute also to EVD control^1^,^2^,^15^. Reducing contacts between patients and nurses may limit HCW exposure to contagious patients for whom EVD diagnosis is missed or delayed.

Chowell *et al.*^7^ reported the strong outcome of early EVD detection during the pre-symptomatic stage. With 65% effectiveness of control measures in these patients, the attack rate in hospital was dramatically reduced below the epidemic threshold. Our results, based on documented contact patterns between individuals, can be interpreted similarly.

It was reported recently^4^ that HCW contributed little to transmission and that community chains of transmission might be controlled by hospitalization. Indeed, a 10% increase of hospitalization rates was associated with 26% reduction of transmission chain length. It was also observed^4^ that a “super-spreading event was due to loosening of controls when the local epidemic was believed to be mostly over”. These very important points suggest that efficient hospital infection control and prevention measures must be implemented and maintained until the outbreaks are declared to have ended.

Patients may be admitted to non-outbreak areas in university or non-university hospitals after various modes of entry into the country, such as by car, train, plane or ship. HCW education and information are needed via epidemiological and clinical data to facilitate early diagnoses or alerts in suspected cases because of direct impact on nosocomial transmission.

All calculations are based on contact matrices provided by effective measurement during care^12^. Previously-reported nosocomial EVD transmission rates^15^, distance at risk of infection^16^ and the stochastic nature of the model have made results more interpretable^11^. We observed a plateau of emergence probability after a transmission probability threshold of 0.03 (Fig. 2). This result suggests that regular increments of SIC cannot be expected when transmission probability increases, which was also reported recently from contact data obtained in various settings by Leventhal *et al.*^17^ for infectious diseases other than EVD.

Some study limitations should be addressed. The observed contact matrices came from an acute geriatric unit and certainly differ from other medical or intensive care wards. We assumed that effectiveness of control measures was 100% as soon as an index case was detected, which may be the ideal scenario but might differed from the actual situation. Then, our estimations could be underestimated. Although we tested contact matrices, which represent well interactions between patients, nurses and physicians, we ignored the heterogeneity of individual behaviours. The analysis did not considered infected materials, bad cleaning of contaminated areas, and manipulation areas (i.e. blood exposure in biological laboratories), in the EVD spreading model. Valid data on that topic were not available. Specific studies on this subject may be needed and integrated in the most parsimonious models including patterns of inter-individual contacts.

These findings underscore EVD emergence potential in hospitals in case of patient admission without specific information and the implementation of specific prevention and control measures, in a European country. Early detection of these patients through epidemiological questioning in triage to preventing their introduction to a unit, as used for a Dutch case of Marburg Hemorrhagic Fever^18^, need to be also implemented.

Nurses are a population for which particular attention is warranted in EVD outbreaks because they are exposed the most to infections^19^. Appropriate EVD education and training of HCW in non-outbreak areas may help to limit EVD risk among patients admitted in the pre-symptomatic stage of EVD.

## Additional Information

**How to cite this article**: Vanhems, P. *et al.* Emergence of Ebola virus disease in a french acute care setting: a simulation study based on documented inter-individual contacts. *Sci. Rep.* **6**, 36301; doi: 10.1038/srep36301 (2016).

**Publisher’s note**: Springer Nature remains neutral with regard to jurisdictional claims in published maps and institutional affiliations.

## Supplementary Material

Supplementary Information

## Figures and Tables

**Table 1:** Description of model parameters with default and varying values.

**Parameter**	**Meaning**	**Value**	**References**
N^PAT^	Number of patients	27	12
N^NUR^	Number of nurses	29	12
N^PHY^	Number of physicians	11	12
*w*_*ij*_	Average number of individuals from group *i* with individuals from group *j*, with 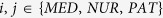	Varied between 5 and 27.3 for *w*_*NUR, PAT*_ (otherwise set according to Table 2)	12
*p*	Transmission probability per infectious contact	Varied between 0.001 and 1	
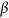 _*ij*_	Transmission coefficient between individuals of group *i* with individuals of group *j*, with 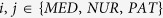	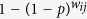	
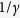	Incubation period	11 days	17
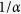	I_1_ duration	Varied between 1 and 3 days (otherwise set at 2 days)	

**Table 2:** Observed contact matrices between patients, nurses and physicians in the model.

**Observed contact matrix**	**Physicians**	**Nurses**	**Patients**
Physicians	97.1	23.2	13.0
Nurses	9.5	98.3	27.3
Patients	4.9	25.4	1.7

Each cell indicates the average number of contacts per day that a physician, nurse or patient (in row) had with other physicians, nurses or patients (in columns). For example, the average number of nurse contacts with patients is 27.3 per day^12^.

**Table 3:** Description of model transitions and their rates.

**Transition**	**Meaning**	**Transition rate**
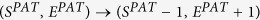	Infection of a patient	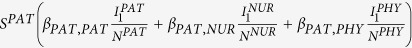
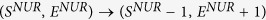	Infection of a nurse	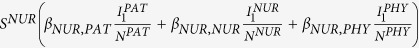
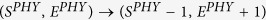	Infection of a physician	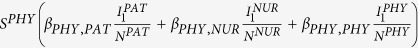
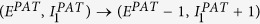	Onset of nonspecific EVD symptoms in a patient	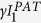
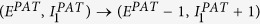	Onset of nonspecific EVD symptoms in a nurse	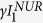
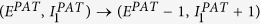	Onset of nonspecific EVD symptoms in a physician	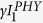
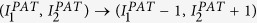	Onset of specific EVD symptoms, detection and isolation of a patient	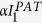
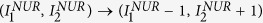	Onset of specific EVD symptoms, detection and isolation of a nurse	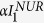
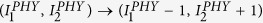	Onset of specific EVD symptoms, detection and isolation of a physician	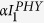

Susceptible (S), exposed (E, i.e., disease incubation), contagious with nonspecific EVD symptoms (I_1_) and contagious with specific EVD symptoms (I_2_).
