# Supplementary material for: Emergence of Ebola virus disease in a french acute care setting: a simulation study based on documented inter-individual contacts
Source: Sci Rep. 2016 Nov 9;6:36301. doi: 10.1038/srep36301 (PMC5101488; doi:10.1038/srep36301)

# Supplementary information

## Emergence of Ebola virus disease in a french acute care setting: a simulation study based on documented inter-individual contacts

Philippe Vanhems[1,2], Rose Von Raesfeldt[3,4,5,6], René Ecochard[3,4,5,6], Nicolas Voirin[3,4,5,6]

[1]Service d'Hygiène, Epidémiologie et Prévention, Hôpital Edouard Herriot, Hospices Civils de Lyon, F-69437 Lyon, France
[2]Laboratoire des Pathogènes Emergents – Fondation Mérieux, Centre International de Recherche en Infectiologie, Institut National de la Santé et de la Recherche Médicale U1111, Centre National de la Recherche Scientifique, UMR5308, Ecole Normale Supérieure de Lyon, Université Claude Bernard Lyon 1, 21, Avenue Tony Garnier, 69007 Lyon, France
[3]Université de Lyon, F-69000 Lyon, France
[4]Université Lyon 1, F-69100 Villeurbanne, France
[5]Service de Biostatistique, Hospices Civils de Lyon, F-69003 Lyon, France
[6]Laboratoire de Biométrie et Biologie Evolutive, Equipe Biostatistique-Santé, Centre National de la Recherche Scientifique, UMR5558, F-69100 Villeurbanne, France

**Emergence probability and number of SIC according to transmission probability: analysis by population (patients, nurses, and physicians)**

Emergence Probability per transission probability

OVERALL( 67 )

------

| | p | pemerg | IC_ALL_mean | IC_ALL_min | IC_ALL_max | pemerg_min | pemerg_max |
|---|---|---|---|---|---|---|---|
| 1 | 0.001 | 0.0730 | 1.061644 | 1 | 3 | 0.0540 | 0.0925 |
| 2 | 0.002 | 0.1350 | 1.207407 | 1 | 5 | 0.1120 | 0.1600 |
| 3 | 0.003 | 0.1695 | 1.244838 | 1 | 4 | 0.1460 | 0.1975 |
| 4 | 0.005 | 0.2465 | 1.397566 | 1 | 8 | 0.2150 | 0.2735 |
| 5 | 0.010 | 0.3975 | 1.713208 | 1 | 9 | 0.3635 | 0.4320 |
| 6 | 0.020 | 0.5285 | 2.315989 | 1 | 15 | 0.4980 | 0.5660 |
| 7 | 0.030 | 0.6175 | 2.651012 | 1 | 18 | 0.5825 | 0.6515 |
| 8 | 0.040 | 0.6630 | 2.972097 | 1 | 21 | 0.6290 | 0.7120 |
| 9 | 0.050 | 0.6970 | 3.428981 | 1 | 23 | 0.6635 | 0.7300 |
| 10 | 0.100 | 0.7350 | 3.960544 | 1 | 22 | 0.7015 | 0.7685 |
| 11 | 0.150 | 0.7850 | 4.347771 | 1 | 32 | 0.7535 | 0.8125 |
| 12 | 0.200 | 0.7965 | 4.577527 | 1 | 27 | 0.7705 | 0.8230 |
| 13 | 0.250 | 0.7750 | 4.628387 | 1 | 35 | 0.7505 | 0.8045 |
| 14 | 0.300 | 0.7885 | 4.715282 | 1 | 32 | 0.7620 | 0.8155 |
| 15 | 0.400 | 0.8165 | 5.008573 | 1 | 36 | 0.7865 | 0.8465 |
| 16 | 0.500 | 0.8220 | 5.304136 | 1 | 34 | 0.7930 | 0.8495 |
| 17 | 0.600 | 0.8085 | 5.507112 | 1 | 37 | 0.7775 | 0.8330 |
| 18 | 0.700 | 0.8175 | 5.507645 | 1 | 36 | 0.7930 | 0.8430 |
| 19 | 0.800 | 0.8115 | 5.507702 | 1 | 31 | 0.7820 | 0.8490 |
| 20 | 0.900 | 0.8375 | 5.533731 | 1 | 35 | 0.8085 | 0.8640 |
| 21 | 1.000 | 0.8315 | 5.580878 | 1 | 35 | 0.8025 | 0.8550 |

NUR( 29 )

------

| | p | pemerg | IC_NUR_mean | IC_NUR_min | IC_NUR_max | pemerg_min | pemerg_max |
|---|---|---|---|---|---|---|---|
| 1 | 0.001 | 0.0585 | 1.059829 | 1 | 3 | 0.0435 | 0.0750 |
| 2 | 0.002 | 0.1105 | 1.162896 | 1 | 3 | 0.0900 | 0.1335 |
| 3 | 0.003 | 0.1430 | 1.202797 | 1 | 4 | 0.1195 | 0.1695 |
| 4 | 0.005 | 0.2075 | 1.308434 | 1 | 5 | 0.1790 | 0.2320 |
| 5 | 0.010 | 0.3380 | 1.559172 | 1 | 9 | 0.3050 | 0.3685 |
| 6 | 0.020 | 0.4730 | 1.988372 | 1 | 10 | 0.4385 | 0.5095 |
| 7 | 0.030 | 0.5560 | 2.186151 | 1 | 12 | 0.5220 | 0.5905 |
| 8 | 0.040 | 0.5975 | 2.330544 | 1 | 12 | 0.5620 | 0.6340 |
| 9 | 0.050 | 0.6315 | 2.639747 | 1 | 13 | 0.5980 | 0.6665 |
| 10 | 0.100 | 0.6640 | 2.798946 | 1 | 13 | 0.6290 | 0.6955 |
| 11 | 0.150 | 0.6960 | 2.966954 | 1 | 16 | 0.6555 | 0.7280 |
| 12 | 0.200 | 0.6930 | 2.998557 | 1 | 16 | 0.6550 | 0.7255 |
| 13 | 0.250 | 0.6635 | 3.004521 | 1 | 21 | 0.6215 | 0.6945 |
| 14 | 0.300 | 0.6795 | 2.924945 | 1 | 16 | 0.6420 | 0.7140 |
| 15 | 0.400 | 0.6940 | 3.031700 | 1 | 18 | 0.6655 | 0.7330 |
| 16 | 0.500 | 0.6990 | 3.055794 | 1 | 18 | 0.6660 | 0.7305 |
| 17 | 0.600 | 0.6700 | 3.097015 | 1 | 18 | 0.6360 | 0.7015 |
| 18 | 0.700 | 0.6740 | 3.018546 | 1 | 16 | 0.6395 | 0.7080 |
| 19 | 0.800 | 0.6675 | 2.962547 | 1 | 16 | 0.6350 | 0.7020 |
| 20 | 0.900 | 0.6785 | 3.000737 | 1 | 16 | 0.6395 | 0.7085 |
| 21 | 1.000 | 0.6905 | 3.039826 | 1 | 18 | 0.6595 | 0.7205 |

PHY( 11 )

------

| | p | pemerg | IC_PHY_mean | IC_PHY_min | IC_PHY_max | pemerg_min | pemerg_max |
|---|---|---|---|---|---|---|---|
| 1 | 0.001 | 0.0110 | 1.045455 | 1 | 2 | 0.0045 | 0.0190 |
| 2 | 0.002 | 0.0220 | 1.045455 | 1 | 2 | 0.0115 | 0.0335 |
| 3 | 0.003 | 0.0265 | 1.056604 | 1 | 2 | 0.0170 | 0.0370 |
| 4 | 0.005 | 0.0535 | 1.037383 | 1 | 2 | 0.0405 | 0.0680 |
| 5 | 0.010 | 0.0895 | 1.201117 | 1 | 5 | 0.0685 | 0.1110 |

|  |  |  |  |  |  |  |  |
|---|---|---|---|---|---|---|---|
| 6 | 0.020 | 0.1540 | 1.250000 | 1 | 5 | 0.1300 | 0.1785 |
| 7 | 0.030 | 0.2195 | 1.282460 | 1 | 5 | 0.1910 | 0.2585 |
| 8 | 0.040 | 0.2670 | 1.380150 | 1 | 8 | 0.2390 | 0.2970 |
| 9 | 0.050 | 0.2980 | 1.483221 | 1 | 7 | 0.2625 | 0.3350 |
| 10 | 0.100 | 0.3845 | 1.574772 | 1 | 7 | 0.3515 | 0.4190 |
| 11 | 0.150 | 0.4210 | 1.743468 | 1 | 8 | 0.3825 | 0.4550 |
| 12 | 0.200 | 0.4435 | 1.706877 | 1 | 7 | 0.4045 | 0.4820 |
| 13 | 0.250 | 0.4305 | 1.738676 | 1 | 7 | 0.3965 | 0.4710 |
| 14 | 0.300 | 0.4330 | 1.717090 | 1 | 8 | 0.3930 | 0.4665 |
| 15 | 0.400 | 0.4355 | 1.748565 | 1 | 8 | 0.3970 | 0.4790 |
| 16 | 0.500 | 0.4785 | 1.717868 | 1 | 8 | 0.4395 | 0.5150 |
| 17 | 0.600 | 0.4580 | 1.791485 | 1 | 7 | 0.4230 | 0.4940 |
| 18 | 0.700 | 0.4475 | 1.760894 | 1 | 9 | 0.4075 | 0.4890 |
| 19 | 0.800 | 0.4590 | 1.696078 | 1 | 8 | 0.4240 | 0.4875 |
| 20 | 0.900 | 0.4500 | 1.647778 | 1 | 8 | 0.4170 | 0.4915 |
| 21 | 1.000 | 0.4390 | 1.668565 | 1 | 7 | 0.4045 | 0.4675 |

PAT( 27 )
------

|  | p | pemerg | IC_PAT_mean | IC_PAT_min | IC_PAT_max | pemerg_min | pemerg_max |
|---|---|---|---|---|---|---|---|
| 1 | 0.001 | 0.0040 | 1.000000 | 1 | 1 | 0.0010 | 0.0100 |
| 2 | 0.002 | 0.0105 | 1.095238 | 1 | 2 | 0.0035 | 0.0170 |
| 3 | 0.003 | 0.0105 | 1.047619 | 1 | 2 | 0.0035 | 0.0175 |
| 4 | 0.005 | 0.0170 | 1.029412 | 1 | 2 | 0.0055 | 0.0275 |
| 5 | 0.010 | 0.0415 | 1.120482 | 1 | 2 | 0.0285 | 0.0575 |
| 6 | 0.020 | 0.0765 | 1.189542 | 1 | 4 | 0.0580 | 0.0940 |
| 7 | 0.030 | 0.1090 | 1.284404 | 1 | 5 | 0.0895 | 0.1340 |
| 8 | 0.040 | 0.1485 | 1.410774 | 1 | 5 | 0.1250 | 0.1780 |
| 9 | 0.050 | 0.1865 | 1.506702 | 1 | 8 | 0.1630 | 0.2195 |
| 10 | 0.100 | 0.2725 | 1.640367 | 1 | 6 | 0.2435 | 0.3025 |
| 11 | 0.150 | 0.3440 | 1.784884 | 1 | 11 | 0.3055 | 0.3810 |
| 12 | 0.200 | 0.4275 | 1.897076 | 1 | 10 | 0.3955 | 0.4660 |
| 13 | 0.250 | 0.4345 | 1.944764 | 1 | 11 | 0.3970 | 0.4745 |
| 14 | 0.300 | 0.4800 | 2.056250 | 1 | 11 | 0.4415 | 0.5185 |
| 15 | 0.400 | 0.5505 | 2.223433 | 1 | 13 | 0.5110 | 0.5850 |
| 16 | 0.500 | 0.5770 | 2.429809 | 1 | 11 | 0.5430 | 0.6095 |
| 17 | 0.600 | 0.6065 | 2.567189 | 1 | 14 | 0.5765 | 0.6395 |
| 18 | 0.700 | 0.6285 | 2.673031 | 1 | 14 | 0.5910 | 0.6550 |
| 19 | 0.800 | 0.6405 | 2.675254 | 1 | 11 | 0.6075 | 0.6780 |
| 20 | 0.900 | 0.6725 | 2.761338 | 1 | 15 | 0.6390 | 0.7070 |
| 21 | 1.000 | 0.6660 | 2.716216 | 1 | 14 | 0.6320 | 0.6970 |

**Variability of emergence probability of EVD in a single hospital ward according to transmission probability and number of nurse (NUR) contacts with patients (PAT) by population**

| | Transmission probability | Daily contacts of NUR with PAT | pemerge_mean_ALL | pemerge_min_ALL | pemerge_max_ALL | pemerge_mean_NUR | pemerge_min_NUR | pemerge_max_NUR | pemerge_mean_PAT | pemerge_min_PAT | pemerge_max_PAT | pemerge_mean_PHY | pemerge_min_PHY | pemerge_max_PHY |
|---|---|---|---|---|---|---|---|---|---|---|---|---|---|---|
| 1 | 0.000 | 0.0 | 0.0000 | 0.0000 | 0.0000 | 0.0000 | 0.0000 | 0.0000 | 0.0000 | 0.0000 | 0.0000 | 0.0000 | 0.0000 | 0.0000 |
| 2 | 0.001 | 27.3 | 0.0730 | 0.0545 | 0.0920 | 0.0585 | 0.0415 | 0.0755 | 0.0040 | 0.0000 | 0.0100 | 0.0110 | 0.0040 | 0.0180 |
| 3 | 0.001 | 20.0 | 0.0540 | 0.0380 | 0.0710 | 0.0395 | 0.0260 | 0.0545 | 0.0020 | 0.0000 | 0.0065 | 0.0135 | 0.0070 | 0.0215 |
| 4 | 0.001 | 15.0 | 0.0490 | 0.0345 | 0.0645 | 0.0280 | 0.0185 | 0.0395 | 0.0075 | 0.0025 | 0.0140 | 0.0140 | 0.0055 | 0.0245 |
| 5 | 0.001 | 10.0 | 0.0470 | 0.0320 | 0.0610 | 0.0275 | 0.0145 | 0.0400 | 0.0065 | 0.0020 | 0.0155 | 0.0140 | 0.0050 | 0.0235 |
| 6 | 0.001 | 5.0 | 0.0240 | 0.0130 | 0.0350 | 0.0090 | 0.0035 | 0.0175 | 0.0035 | 0.0000 | 0.0080 | 0.0115 | 0.0045 | 0.0225 |
| 7 | 0.002 | 27.3 | 0.1350 | 0.1130 | 0.1580 | 0.1105 | 0.0880 | 0.1330 | 0.0105 | 0.0035 | 0.0185 | 0.0220 | 0.0120 | 0.0330 |
| 8 | 0.002 | 20.0 | 0.1010 | 0.0800 | 0.1195 | 0.0760 | 0.0575 | 0.0940 | 0.0060 | 0.0015 | 0.0115 | 0.0255 | 0.0150 | 0.0365 |
| 9 | 0.002 | 15.0 | 0.0755 | 0.0535 | 0.0985 | 0.0545 | 0.0395 | 0.0700 | 0.0080 | 0.0030 | 0.0155 | 0.0165 | 0.0070 | 0.0275 |
| 10 | 0.002 | 10.0 | 0.0720 | 0.0555 | 0.0945 | 0.0525 | 0.0380 | 0.0705 | 0.0050 | 0.0010 | 0.0100 | 0.0175 | 0.0100 | 0.0300 |
| 11 | 0.002 | 5.0 | 0.0435 | 0.0275 | 0.0575 | 0.0235 | 0.0125 | 0.0360 | 0.0055 | 0.0010 | 0.0120 | 0.0160 | 0.0065 | 0.0285 |
| 12 | 0.003 | 27.3 | 0.1695 | 0.1440 | 0.1975 | 0.1430 | 0.1180 | 0.1655 | 0.0105 | 0.0050 | 0.0175 | 0.0265 | 0.0175 | 0.0405 |
| 13 | 0.003 | 20.0 | 0.1475 | 0.1215 | 0.1695 | 0.1180 | 0.0960 | 0.1380 | 0.0075 | 0.0025 | 0.0140 | 0.0310 | 0.0200 | 0.0440 |
| 14 | 0.003 | 15.0 | 0.1270 | 0.1070 | 0.1505 | 0.0910 | 0.0725 | 0.1095 | 0.0090 | 0.0030 | 0.0170 | 0.0315 | 0.0205 | 0.0440 |
| 15 | 0.003 | 10.0 | 0.0900 | 0.0705 | 0.1095 | 0.0595 | 0.0435 | 0.0765 | 0.0090 | 0.0040 | 0.0155 | 0.0290 | 0.0185 | 0.0415 |
| 16 | 0.003 | 5.0 | 0.0645 | 0.0480 | 0.0840 | 0.0320 | 0.0175 | 0.0425 | 0.0110 | 0.0040 | 0.0205 | 0.0255 | 0.0155 | 0.0365 |
| 17 | 0.005 | 27.3 | 0.2465 | 0.2135 | 0.2750 | 0.2075 | 0.1800 | 0.2395 | 0.0170 | 0.0085 | 0.0275 | 0.0535 | 0.0380 | 0.0775 |
| 18 | 0.005 | 20.0 | 0.2150 | 0.1875 | 0.2490 | 0.1665 | 0.1405 | 0.1930 | 0.0210 | 0.0120 | 0.0315 | 0.0510 | 0.0350 | 0.0670 |
| 19 | 0.005 | 15.0 | 0.1900 | 0.1570 | 0.2190 | 0.1405 | 0.1165 | 0.1625 | 0.0200 | 0.0110 | 0.0365 | 0.0475 | 0.0330 | 0.0650 |
| 20 | 0.005 | 10.0 | 0.1440 | 0.1150 | 0.1690 | 0.1025 | 0.0770 | 0.1245 | 0.0160 | 0.0075 | 0.0265 | 0.0425 | 0.0260 | 0.0555 |
| 21 | 0.005 | 5.0 | 0.1090 | 0.0835 | 0.1310 | 0.0575 | 0.0405 | 0.0740 | 0.0145 | 0.0065 | 0.0235 | 0.0485 | 0.0310 | 0.0635 |
| 22 | 0.010 | 27.3 | 0.3975 | 0.3610 | 0.4375 | 0.3380 | 0.3035 | 0.3745 | 0.0415 | 0.0260 | 0.0555 | 0.0895 | 0.0710 | 0.1125 |
| 23 | 0.010 | 20.0 | 0.3310 | 0.2930 | 0.3660 | 0.2740 | 0.2395 | 0.3060 | 0.0425 | 0.0255 | 0.0585 | 0.0890 | 0.0695 | 0.1085 |
| 24 | 0.010 | 15.0 | 0.2820 | 0.2420 | 0.3145 | 0.2090 | 0.1780 | 0.2375 | 0.0390 | 0.0250 | 0.0545 | 0.0845 | 0.0635 | 0.1045 |
| 25 | 0.010 | 10.0 | 0.2470 | 0.2205 | 0.2800 | 0.1620 | 0.1375 | 0.1925 | 0.0360 | 0.0240 | 0.0535 | 0.0980 | 0.0750 | 0.1210 |
| 26 | 0.010 | 5.0 | 0.1810 | 0.1535 | 0.2120 | 0.0930 | 0.0725 | 0.1145 | 0.0340 | 0.0225 | 0.0480 | 0.0810 | 0.0630 | 0.0990 |
| 27 | 0.020 | 27.3 | 0.5285 | 0.4980 | 0.5630 | 0.4730 | 0.4340 | 0.5160 | 0.0765 | 0.0605 | 0.0945 | 0.1540 | 0.1255 | 0.1805 |
| 28 | 0.020 | 20.0 | 0.4985 | 0.4615 | 0.5275 | 0.4285 | 0.3910 | 0.4600 | 0.0835 | 0.0630 | 0.1010 | 0.1670 | 0.1445 | 0.1980 |
| 29 | 0.020 | 15.0 | 0.4480 | 0.4075 | 0.4880 | 0.3590 | 0.3220 | 0.3975 | 0.0825 | 0.0625 | 0.1040 | 0.1595 | 0.1350 | 0.1825 |
| 30 | 0.020 | 10.0 | 0.4055 | 0.3580 | 0.4370 | 0.2985 | 0.2640 | 0.3270 | 0.0745 | 0.0525 | 0.0975 | 0.1670 | 0.1445 | 0.1945 |
| 31 | 0.020 | 5.0 | 0.3140 | 0.2695 | 0.3470 | 0.1720 | 0.1445 | 0.2020 | 0.0680 | 0.0505 | 0.0885 | 0.1490 | 0.1255 | 0.1700 |
| 32 | 0.030 | 27.3 | 0.6175 | 0.5800 | 0.6560 | 0.5560 | 0.5110 | 0.5920 | 0.1090 | 0.0845 | 0.1310 | 0.2195 | 0.1880 | 0.2535 |
| 33 | 0.030 | 20.0 | 0.5610 | 0.5285 | 0.6050 | 0.4785 | 0.4465 | 0.5185 | 0.1155 | 0.0940 | 0.1415 | 0.2070 | 0.1800 | 0.2355 |
| 34 | 0.030 | 15.0 | 0.5380 | 0.5070 | 0.5710 | 0.4415 | 0.4045 | 0.4755 | 0.1125 | 0.0880 | 0.1370 | 0.2245 | 0.1915 | 0.2545 |
| 35 | 0.030 | 10.0 | 0.4705 | 0.4390 | 0.5020 | 0.3495 | 0.3195 | 0.3825 | 0.0985 | 0.0755 | 0.1215 | 0.2110 | 0.1830 | 0.2400 |
| 36 | 0.030 | 5.0 | 0.3975 | 0.3600 | 0.4330 | 0.2460 | 0.2130 | 0.2770 | 0.1045 | 0.0825 | 0.1285 | 0.2040 | 0.1790 | 0.2395 |
| 37 | 0.040 | 27.3 | 0.6630 | 0.6280 | 0.6955 | 0.5975 | 0.5660 | 0.6350 | 0.1485 | 0.1265 | 0.1725 | 0.2670 | 0.2365 | 0.3035 |
| 38 | 0.040 | 20.0 | 0.6215 | 0.5900 | 0.6590 | 0.5415 | 0.5045 | 0.5740 | 0.1360 | 0.1145 | 0.1640 | 0.2460 | 0.2140 | 0.2800 |
| 39 | 0.040 | 15.0 | 0.5975 | 0.5590 | 0.6310 | 0.5025 | 0.4720 | 0.5420 | 0.1605 | 0.1305 | 0.1910 | 0.2435 | 0.2145 | 0.2755 |
| 40 | 0.040 | 10.0 | 0.5575 | 0.5235 | 0.5915 | 0.4200 | 0.3865 | 0.4545 | 0.1375 | 0.1170 | 0.1635 | 0.2820 | 0.2490 | 0.3180 |
| 41 | 0.040 | 5.0 | 0.4720 | 0.4310 | 0.5030 | 0.2970 | 0.2645 | 0.3350 | 0.1285 | 0.1065 | 0.1535 | 0.2460 | 0.2175 | 0.2800 |
| 42 | 0.050 | 27.3 | 0.6970 | 0.6635 | 0.7310 | 0.6315 | 0.5985 | 0.6625 | 0.1865 | 0.1545 | 0.2125 | 0.2980 | 0.2690 | 0.3295 |
| 43 | 0.050 | 20.0 | 0.6550 | 0.6250 | 0.6930 | 0.5825 | 0.5460 | 0.6230 | 0.1890 | 0.1650 | 0.2295 | 0.2950 | 0.2620 | 0.3250 |
| 44 | 0.050 | 15.0 | 0.6350 | 0.6035 | 0.6760 | 0.5410 | 0.5045 | 0.5830 | 0.1900 | 0.1650 | 0.2165 | 0.2905 | 0.2565 | 0.3330 |
| 45 | 0.050 | 10.0 | 0.5915 | 0.5575 | 0.6305 | 0.4645 | 0.4300 | 0.4990 | 0.1740 | 0.1475 | 0.2060 | 0.2985 | 0.2655 | 0.3365 |
| 46 | 0.050 | 5.0 | 0.5320 | 0.4975 | 0.5665 | 0.3500 | 0.3150 | 0.3830 | 0.1735 | 0.1440 | 0.2010 | 0.3035 | 0.2725 | 0.3375 |
| 47 | 0.100 | 27.3 | 0.7350 | 0.6935 | 0.7615 | 0.6640 | 0.6290 | 0.6995 | 0.2725 | 0.2370 | 0.3075 | 0.3845 | 0.3470 | 0.4170 |
| 48 | 0.100 | 20.0 | 0.7580 | 0.7265 | 0.7915 | 0.6775 | 0.6460 | 0.7180 | 0.3070 | 0.2705 | 0.3360 | 0.4015 | 0.3610 | 0.4370 |
| 49 | 0.100 | 15.0 | 0.7315 | 0.6985 | 0.7620 | 0.6425 | 0.6095 | 0.6720 | 0.2890 | 0.2585 | 0.3215 | 0.3935 | 0.3585 | 0.4350 |
| 50 | 0.100 | 10.0 | 0.6945 | 0.6615 | 0.7225 | 0.5785 | 0.5440 | 0.6095 | 0.2720 | 0.2360 | 0.3070 | 0.3830 | 0.3485 | 0.4180 |
| 51 | 0.100 | 5.0 | 0.6435 | 0.6060 | 0.6765 | 0.4805 | 0.4465 | 0.5205 | 0.2645 | 0.2350 | 0.3030 | 0.3810 | 0.3435 | 0.4160 |
| 52 | 0.150 | 27.3 | 0.7850 | 0.7555 | 0.8120 | 0.6960 | 0.6620 | 0.7255 | 0.3440 | 0.3090 | 0.3840 | 0.4210 | 0.3910 | 0.4585 |
| 53 | 0.150 | 20.0 | 0.7635 | 0.7330 | 0.7895 | 0.6690 | 0.6350 | 0.7000 | 0.3510 | 0.3155 | 0.3860 | 0.4195 | 0.3845 | 0.4530 |
| 54 | 0.150 | 15.0 | 0.7725 | 0.7405 | 0.8105 | 0.6725 | 0.6310 | 0.7115 | 0.3590 | 0.3215 | 0.3950 | 0.4260 | 0.3940 | 0.4630 |
| 55 | 0.150 | 10.0 | 0.7255 | 0.6900 | 0.7560 | 0.6270 | 0.5960 | 0.6690 | 0.3445 | 0.3040 | 0.3745 | 0.4085 | 0.3765 | 0.4445 |
| 56 | 0.150 | 5.0 | 0.7140 | 0.6795 | 0.7430 | 0.5525 | 0.5210 | 0.5820 | 0.3430 | 0.3100 | 0.3765 | 0.4360 | 0.3990 | 0.4720 |
| 57 | 0.200 | 27.3 | 0.7965 | 0.7660 | 0.8275 | 0.6930 | 0.6645 | 0.7295 | 0.4275 | 0.3930 | 0.4605 | 0.4435 | 0.4070 | 0.4820 |
| 58 | 0.200 | 20.0 | 0.7765 | 0.7440 | 0.8020 | 0.6795 | 0.6420 | 0.7100 | 0.4085 | 0.3725 | 0.4400 | 0.4335 | 0.4005 | 0.4685 |
| 59 | 0.200 | 15.0 | 0.7720 | 0.7280 | 0.7990 | 0.6680 | 0.6285 | 0.7010 | 0.4020 | 0.3610 | 0.4350 | 0.4330 | 0.3940 | 0.4655 |
| 60 | 0.200 | 10.0 | 0.7760 | 0.7475 | 0.8080 | 0.6635 | 0.6275 | 0.6955 | 0.4115 | 0.3745 | 0.4435 | 0.4545 | 0.4185 | 0.4920 |
| 61 | 0.200 | 5.0 | 0.7390 | 0.7095 | 0.7760 | 0.5920 | 0.5535 | 0.6400 | 0.3925 | 0.3585 | 0.4210 | 0.4355 | 0.4010 | 0.4710 |
| 62 | 0.250 | 27.3 | 0.7750 | 0.7400 | 0.8065 | 0.6635 | 0.6240 | 0.6960 | 0.4345 | 0.3995 | 0.4690 | 0.4305 | 0.3960 | 0.4695 |
| 63 | 0.250 | 20.0 | 0.7835 | 0.7525 | 0.8175 | 0.6800 | 0.6460 | 0.7080 | 0.4530 | 0.4110 | 0.4850 | 0.4530 | 0.4205 | 0.4925 |
| 64 | 0.250 | 15.0 | 0.7860 | 0.7610 | 0.8220 | 0.6775 | 0.6470 | 0.7200 | 0.4280 | 0.3960 | 0.4625 | 0.4355 | 0.3925 | 0.4710 |
| 65 | 0.250 | 10.0 | 0.7800 | 0.7485 | 0.8095 | 0.6630 | 0.6300 | 0.6965 | 0.4510 | 0.4125 | 0.4815 | 0.4370 | 0.3970 | 0.4805 |
| 66 | 0.250 | 5.0 | 0.7525 | 0.7245 | 0.7795 | 0.6125 | 0.5720 | 0.6505 | 0.4365 | 0.4045 | 0.4715 | 0.4200 | 0.3895 | 0.4570 |
| 67 | 0.300 | 27.3 | 0.7885 | 0.7580 | 0.8135 | 0.6795 | 0.6520 | 0.7110 | 0.4800 | 0.4450 | 0.5230 | 0.4330 | 0.3975 | 0.4675 |
| 68 | 0.300 | 20.0 | 0.7780 | 0.7455 | 0.8070 | 0.6710 | 0.6355 | 0.7015 | 0.4885 | 0.4560 | 0.5235 | 0.4510 | 0.4185 | 0.4905 |
| 69 | 0.300 | 15.0 | 0.8020 | 0.7765 | 0.8280 | 0.6830 | 0.6465 | 0.7135 | 0.4835 | 0.4390 | 0.5220 | 0.4495 | 0.4140 | 0.4825 |
| 70 | 0.300 | 10.0 | 0.7900 | 0.7605 | 0.8180 | 0.6720 | 0.6395 | 0.7040 | 0.4710 | 0.4355 | 0.5040 | 0.4540 | 0.4150 | 0.4940 |
| 71 | 0.300 | 5.0 | 0.7755 | 0.7450 | 0.8015 | 0.6415 | 0.6040 | 0.6805 | 0.4580 | 0.4210 | 0.4960 | 0.4335 | 0.3995 | 0.4645 |
| 72 | 0.400 | 27.3 | 0.8165 | 0.7880 | 0.8415 | 0.6940 | 0.6620 | 0.7230 | 0.5505 | 0.5180 | 0.5835 | 0.4355 | 0.3995 | 0.4745 |
| 73 | 0.400 | 20.0 | 0.7980 | 0.7620 | 0.8250 | 0.6760 | 0.6405 | 0.7130 | 0.5275 | 0.4880 | 0.5580 | 0.4475 | 0.4090 | 0.4815 |
| 74 | 0.400 | 15.0 | 0.7995 | 0.7715 | 0.8265 | 0.6725 | 0.6355 | 0.7040 | 0.5545 | 0.5105 | 0.5880 | 0.4295 | 0.3970 | 0.4620 |

| | | | | | | | | | | | | | | |
|---|---|---|---|---|---|---|---|---|---|---|---|---|---|---|
| 75 | 0.400 | 10.0 | 0.8130 | 0.7875 | 0.8415 | 0.6890 | 0.6585 | 0.7290 | 0.5490 | 0.5175 | 0.5920 | 0.4360 | 0.3915 | 0.4725 |
| 76 | 0.400 | 5.0 | 0.8025 | 0.7730 | 0.8360 | 0.6785 | 0.6390 | 0.7110 | 0.5185 | 0.4840 | 0.5635 | 0.4520 | 0.4165 | 0.4830 |
| 77 | 0.500 | 27.3 | 0.8220 | 0.7870 | 0.8470 | 0.6990 | 0.6630 | 0.7300 | 0.5770 | 0.5465 | 0.6120 | 0.4785 | 0.4460 | 0.5195 |
| 78 | 0.500 | 20.0 | 0.8130 | 0.7845 | 0.8380 | 0.6920 | 0.6605 | 0.7225 | 0.5905 | 0.5575 | 0.6270 | 0.4590 | 0.4220 | 0.4930 |
| 79 | 0.500 | 15.0 | 0.8230 | 0.7940 | 0.8540 | 0.6915 | 0.6530 | 0.7260 | 0.5805 | 0.5485 | 0.6165 | 0.4565 | 0.4255 | 0.4935 |
| 80 | 0.500 | 10.0 | 0.8170 | 0.7870 | 0.8475 | 0.6820 | 0.6500 | 0.7180 | 0.5965 | 0.5560 | 0.6295 | 0.4555 | 0.4135 | 0.4905 |
| 81 | 0.500 | 5.0 | 0.8120 | 0.7825 | 0.8355 | 0.6755 | 0.6420 | 0.7090 | 0.5990 | 0.5630 | 0.6320 | 0.4520 | 0.4170 | 0.4885 |
| 82 | 0.600 | 27.3 | 0.8085 | 0.7800 | 0.8330 | 0.6700 | 0.6315 | 0.6980 | 0.6065 | 0.5725 | 0.6385 | 0.4580 | 0.4190 | 0.4905 |
| 83 | 0.600 | 20.0 | 0.8210 | 0.7920 | 0.8500 | 0.6775 | 0.6470 | 0.7075 | 0.6090 | 0.5780 | 0.6460 | 0.4460 | 0.4025 | 0.4835 |
| 84 | 0.600 | 15.0 | 0.8195 | 0.7910 | 0.8455 | 0.6965 | 0.6660 | 0.7265 | 0.6065 | 0.5680 | 0.6430 | 0.4495 | 0.4095 | 0.4930 |
| 85 | 0.600 | 10.0 | 0.8135 | 0.7855 | 0.8405 | 0.6760 | 0.6405 | 0.7065 | 0.5985 | 0.5610 | 0.6370 | 0.4430 | 0.4070 | 0.4760 |
| 86 | 0.600 | 5.0 | 0.8110 | 0.7815 | 0.8410 | 0.6820 | 0.6470 | 0.7180 | 0.6010 | 0.5665 | 0.6480 | 0.4485 | 0.4085 | 0.4830 |
| 87 | 0.700 | 27.3 | 0.8175 | 0.7895 | 0.8500 | 0.6740 | 0.6415 | 0.7095 | 0.6285 | 0.5895 | 0.6760 | 0.4475 | 0.4060 | 0.4820 |
| 88 | 0.700 | 20.0 | 0.8205 | 0.7925 | 0.8495 | 0.6730 | 0.6425 | 0.7035 | 0.6210 | 0.5840 | 0.6590 | 0.4390 | 0.4005 | 0.4745 |
| 89 | 0.700 | 15.0 | 0.8330 | 0.8015 | 0.8550 | 0.6940 | 0.6580 | 0.7290 | 0.6305 | 0.5970 | 0.6645 | 0.4475 | 0.4105 | 0.4805 |
| 90 | 0.700 | 10.0 | 0.8105 | 0.7775 | 0.8370 | 0.6485 | 0.6120 | 0.6855 | 0.6265 | 0.5920 | 0.6555 | 0.4500 | 0.4005 | 0.4830 |
| 91 | 0.700 | 5.0 | 0.8290 | 0.8020 | 0.8600 | 0.6865 | 0.6525 | 0.7220 | 0.6385 | 0.5965 | 0.6865 | 0.4505 | 0.4165 | 0.4870 |
| 92 | 0.800 | 27.3 | 0.8115 | 0.7830 | 0.8390 | 0.6675 | 0.6330 | 0.6975 | 0.6405 | 0.6085 | 0.6765 | 0.4590 | 0.4245 | 0.4960 |
| 93 | 0.800 | 20.0 | 0.8050 | 0.7790 | 0.8420 | 0.6525 | 0.6175 | 0.6910 | 0.6225 | 0.5930 | 0.6560 | 0.4150 | 0.3830 | 0.4480 |
| 94 | 0.800 | 15.0 | 0.8145 | 0.7900 | 0.8405 | 0.6710 | 0.6390 | 0.7055 | 0.6450 | 0.6085 | 0.6800 | 0.4425 | 0.4050 | 0.4765 |
| 95 | 0.800 | 10.0 | 0.8290 | 0.7940 | 0.8560 | 0.6825 | 0.6450 | 0.7165 | 0.6500 | 0.6135 | 0.6860 | 0.4525 | 0.4140 | 0.4890 |
| 96 | 0.800 | 5.0 | 0.8250 | 0.7995 | 0.8505 | 0.6735 | 0.6375 | 0.7005 | 0.6440 | 0.6145 | 0.6750 | 0.4465 | 0.4080 | 0.4885 |
| 97 | 0.900 | 27.3 | 0.8375 | 0.8055 | 0.8655 | 0.6785 | 0.6405 | 0.7140 | 0.6725 | 0.6415 | 0.7065 | 0.4500 | 0.4145 | 0.4835 |
| 98 | 0.900 | 20.0 | 0.8125 | 0.7890 | 0.8400 | 0.6590 | 0.6265 | 0.6955 | 0.6500 | 0.6045 | 0.6885 | 0.4500 | 0.4140 | 0.4850 |
| 99 | 0.900 | 15.0 | 0.8200 | 0.7905 | 0.8480 | 0.6875 | 0.6560 | 0.7170 | 0.6465 | 0.6120 | 0.6765 | 0.4445 | 0.4100 | 0.4755 |
| 100 | 0.900 | 10.0 | 0.8245 | 0.7980 | 0.8540 | 0.6585 | 0.6250 | 0.6890 | 0.6515 | 0.6110 | 0.6830 | 0.4540 | 0.4200 | 0.4890 |
| 101 | 0.900 | 5.0 | 0.8310 | 0.8085 | 0.8550 | 0.6845 | 0.6525 | 0.7130 | 0.6555 | 0.6140 | 0.6880 | 0.4585 | 0.4220 | 0.4950 |
| 102 | 1.000 | 27.3 | 0.8315 | 0.7985 | 0.8600 | 0.6905 | 0.6565 | 0.7280 | 0.6660 | 0.6350 | 0.6975 | 0.4390 | 0.4025 | 0.4790 |
| 103 | 1.000 | 20.0 | 0.8295 | 0.7960 | 0.8535 | 0.6980 | 0.6615 | 0.7350 | 0.6630 | 0.6305 | 0.6955 | 0.4530 | 0.4175 | 0.4860 |
| 104 | 1.000 | 15.0 | 0.8365 | 0.8060 | 0.8605 | 0.7045 | 0.6760 | 0.7380 | 0.6670 | 0.6310 | 0.6965 | 0.4560 | 0.4175 | 0.4880 |
| 105 | 1.000 | 10.0 | 0.8200 | 0.7895 | 0.8480 | 0.6645 | 0.6320 | 0.6980 | 0.6540 | 0.6220 | 0.6820 | 0.4470 | 0.4165 | 0.4800 |
| 106 | 1.000 | 5.0 | 0.8180 | 0.7900 | 0.8420 | 0.6820 | 0.6495 | 0.7170 | 0.6505 | 0.6120 | 0.6855 | 0.4435 | 0.4095 | 0.4740 |

**Variability of emergence probability of EVD in a single hospital ward according to transmission probability and duration of the dry phase of the index case.**

| | Transmission probability | Dry phase duration | pemerge_mean_ALL | pemerge_min_ALL | pemerge_max_ALL | pemerge_mean_NUR | pemerge_min_NUR | pemerge_max_NUR | pemerge_mean_PAT | pemerge_min_PAT | pemerge_max_PAT | pemerge_mean_PHY | pemerge_min_PHY | pemerge_max_PHY |
|---|---|---|---|---|---|---|---|---|---|---|---|---|---|---|
| 1 | 0.000 | 0.0 | 0.0000 | 0.0000 | 0.0000 | 0.0000 | 0.0000 | 0.0000 | 0.0000 | 0.0000 | 0.0000 | 0.0000 | 0.0000 | 0.0000 |
| 2 | 0.001 | 1.0 | 0.0410 | 0.0285 | 0.0565 | 0.0330 | 0.0200 | 0.0485 | 0.0025 | 0.0000 | 0.0080 | 0.0060 | 0.0010 | 0.0130 |
| 3 | 0.001 | 1.5 | 0.0440 | 0.0280 | 0.0590 | 0.0355 | 0.0200 | 0.0485 | 0.0040 | 0.0005 | 0.0090 | 0.0060 | 0.0015 | 0.0125 |
| 4 | 0.001 | 2.0 | 0.0810 | 0.0610 | 0.1045 | 0.0695 | 0.0520 | 0.0895 | 0.0025 | 0.0000 | 0.0070 | 0.0105 | 0.0040 | 0.0175 |
| 5 | 0.001 | 2.5 | 0.0825 | 0.0620 | 0.1015 | 0.0655 | 0.0505 | 0.0815 | 0.0060 | 0.0015 | 0.0115 | 0.0150 | 0.0080 | 0.0245 |
| 6 | 0.001 | 3.0 | 0.0910 | 0.0720 | 0.1080 | 0.0745 | 0.0575 | 0.0930 | 0.0065 | 0.0015 | 0.0125 | 0.0130 | 0.0045 | 0.0220 |
| 7 | 0.002 | 1.0 | 0.0600 | 0.0430 | 0.0780 | 0.0500 | 0.0355 | 0.0655 | 0.0025 | 0.0000 | 0.0080 | 0.0085 | 0.0035 | 0.0155 |
| 8 | 0.002 | 1.5 | 0.1020 | 0.0805 | 0.1220 | 0.0875 | 0.0680 | 0.1050 | 0.0035 | 0.0005 | 0.0085 | 0.0160 | 0.0075 | 0.0250 |
| 9 | 0.002 | 2.0 | 0.1330 | 0.1110 | 0.1570 | 0.1095 | 0.0910 | 0.1290 | 0.0100 | 0.0035 | 0.0190 | 0.0220 | 0.0125 | 0.0320 |
| 10 | 0.002 | 2.5 | 0.1510 | 0.1265 | 0.1740 | 0.1210 | 0.1010 | 0.1420 | 0.0140 | 0.0060 | 0.0220 | 0.0245 | 0.0140 | 0.0345 |
| 11 | 0.002 | 3.0 | 0.1725 | 0.1445 | 0.2030 | 0.1415 | 0.1175 | 0.1680 | 0.0110 | 0.0040 | 0.0190 | 0.0355 | 0.0245 | 0.0520 |
| 12 | 0.003 | 1.0 | 0.0880 | 0.0680 | 0.1080 | 0.0730 | 0.0545 | 0.0905 | 0.0030 | 0.0000 | 0.0075 | 0.0150 | 0.0070 | 0.0260 |
| 13 | 0.003 | 1.5 | 0.1265 | 0.1030 | 0.1525 | 0.1075 | 0.0840 | 0.1300 | 0.0065 | 0.0015 | 0.0130 | 0.0195 | 0.0090 | 0.0315 |
| 14 | 0.003 | 2.0 | 0.1850 | 0.1570 | 0.2085 | 0.1510 | 0.1230 | 0.1775 | 0.0125 | 0.0050 | 0.0225 | 0.0370 | 0.0230 | 0.0495 |
| 15 | 0.003 | 2.5 | 0.2080 | 0.1790 | 0.2345 | 0.1745 | 0.1485 | 0.2045 | 0.0145 | 0.0065 | 0.0230 | 0.0375 | 0.0225 | 0.0550 |
| 16 | 0.003 | 3.0 | 0.2500 | 0.2210 | 0.2810 | 0.2110 | 0.1800 | 0.2445 | 0.0200 | 0.0110 | 0.0305 | 0.0445 | 0.0275 | 0.0625 |
| 17 | 0.005 | 1.0 | 0.1390 | 0.1125 | 0.1670 | 0.1130 | 0.0915 | 0.1375 | 0.0075 | 0.0020 | 0.0135 | 0.0240 | 0.0140 | 0.0355 |
| 18 | 0.005 | 1.5 | 0.2155 | 0.1875 | 0.2470 | 0.1780 | 0.1540 | 0.2105 | 0.0155 | 0.0065 | 0.0265 | 0.0430 | 0.0300 | 0.0585 |
| 19 | 0.005 | 2.0 | 0.2690 | 0.2370 | 0.2955 | 0.2270 | 0.1965 | 0.2600 | 0.0210 | 0.0095 | 0.0315 | 0.0440 | 0.0295 | 0.0620 |
| 20 | 0.005 | 2.5 | 0.2990 | 0.2620 | 0.3365 | 0.2535 | 0.2160 | 0.2890 | 0.0310 | 0.0170 | 0.0470 | 0.0535 | 0.0385 | 0.0710 |
| 21 | 0.005 | 3.0 | 0.3460 | 0.3030 | 0.3790 | 0.2985 | 0.2660 | 0.3270 | 0.0360 | 0.0235 | 0.0525 | 0.0650 | 0.0470 | 0.0850 |
| 22 | 0.010 | 1.0 | 0.2625 | 0.2285 | 0.2905 | 0.2255 | 0.1970 | 0.2535 | 0.0180 | 0.0100 | 0.0285 | 0.0460 | 0.0315 | 0.0610 |
| 23 | 0.010 | 1.5 | 0.3295 | 0.2920 | 0.3630 | 0.2825 | 0.2470 | 0.3165 | 0.0335 | 0.0210 | 0.0470 | 0.0780 | 0.0580 | 0.0980 |
| 24 | 0.010 | 2.0 | 0.3790 | 0.3485 | 0.4115 | 0.3255 | 0.2960 | 0.3605 | 0.0430 | 0.0265 | 0.0575 | 0.0945 | 0.0745 | 0.1150 |
| 25 | 0.010 | 2.5 | 0.4585 | 0.4235 | 0.4900 | 0.4015 | 0.3600 | 0.4370 | 0.0525 | 0.0375 | 0.0690 | 0.1150 | 0.0920 | 0.1380 |
| 26 | 0.010 | 3.0 | 0.5000 | 0.4600 | 0.5300 | 0.4410 | 0.4040 | 0.4710 | 0.0715 | 0.0535 | 0.0910 | 0.1410 | 0.1150 | 0.1710 |
| 27 | 0.020 | 1.0 | 0.3540 | 0.3275 | 0.3875 | 0.3010 | 0.2730 | 0.3325 | 0.0385 | 0.0230 | 0.0535 | 0.0770 | 0.0560 | 0.0950 |
| 28 | 0.020 | 1.5 | 0.4565 | 0.4145 | 0.4945 | 0.4035 | 0.3630 | 0.4355 | 0.0675 | 0.0500 | 0.0845 | 0.1195 | 0.0965 | 0.1470 |
| 29 | 0.020 | 2.0 | 0.5300 | 0.4975 | 0.5575 | 0.4765 | 0.4425 | 0.5065 | 0.0905 | 0.0720 | 0.1115 | 0.1500 | 0.1250 | 0.1805 |
| 30 | 0.020 | 2.5 | 0.5960 | 0.5645 | 0.6350 | 0.5335 | 0.4995 | 0.5705 | 0.1135 | 0.0950 | 0.1370 | 0.1930 | 0.1635 | 0.2190 |
| 31 | 0.020 | 3.0 | 0.6370 | 0.6050 | 0.6740 | 0.5850 | 0.5435 | 0.6215 | 0.1465 | 0.1200 | 0.1750 | 0.2250 | 0.1920 | 0.2570 |
| 32 | 0.030 | 1.0 | 0.4490 | 0.4105 | 0.4875 | 0.3830 | 0.3420 | 0.4125 | 0.0540 | 0.0390 | 0.0705 | 0.1290 | 0.0985 | 0.1515 |
| 33 | 0.030 | 1.5 | 0.5235 | 0.4880 | 0.5590 | 0.4575 | 0.4235 | 0.4885 | 0.0785 | 0.0580 | 0.1010 | 0.1570 | 0.1345 | 0.1800 |
| 34 | 0.030 | 2.0 | 0.5945 | 0.5615 | 0.6260 | 0.5270 | 0.4900 | 0.5580 | 0.1075 | 0.0865 | 0.1270 | 0.1990 | 0.1710 | 0.2305 |
| 35 | 0.030 | 2.5 | 0.6525 | 0.6205 | 0.6910 | 0.5955 | 0.5655 | 0.6320 | 0.1450 | 0.1180 | 0.1715 | 0.2415 | 0.2140 | 0.2705 |
| 36 | 0.030 | 3.0 | 0.6845 | 0.6500 | 0.7105 | 0.6320 | 0.5935 | 0.6635 | 0.1755 | 0.1465 | 0.2100 | 0.2775 | 0.2485 | 0.3165 |
| 37 | 0.040 | 1.0 | 0.4700 | 0.4375 | 0.5115 | 0.4035 | 0.3705 | 0.4465 | 0.0615 | 0.0460 | 0.0795 | 0.1415 | 0.1190 | 0.1710 |
| 38 | 0.040 | 1.5 | 0.5915 | 0.5545 | 0.6250 | 0.5255 | 0.4860 | 0.5645 | 0.1030 | 0.0805 | 0.1220 | 0.2055 | 0.1760 | 0.2350 |
| 39 | 0.040 | 2.0 | 0.6745 | 0.6415 | 0.7185 | 0.6125 | 0.5745 | 0.6480 | 0.1555 | 0.1285 | 0.1890 | 0.2545 | 0.2215 | 0.2820 |
| 40 | 0.040 | 2.5 | 0.7200 | 0.6810 | 0.7530 | 0.6495 | 0.6130 | 0.6835 | 0.1800 | 0.1550 | 0.2075 | 0.3075 | 0.2785 | 0.3415 |
| 41 | 0.040 | 3.0 | 0.7330 | 0.6975 | 0.7645 | 0.6815 | 0.6395 | 0.7155 | 0.2335 | 0.2030 | 0.2730 | 0.3475 | 0.3140 | 0.3955 |
| 42 | 0.050 | 1.0 | 0.5110 | 0.4740 | 0.5450 | 0.4415 | 0.4120 | 0.4765 | 0.0755 | 0.0575 | 0.0955 | 0.1675 | 0.1405 | 0.1950 |
| 43 | 0.050 | 1.5 | 0.6135 | 0.5750 | 0.6460 | 0.5390 | 0.4970 | 0.5705 | 0.1315 | 0.1035 | 0.1585 | 0.2175 | 0.1900 | 0.2460 |
| 44 | 0.050 | 2.0 | 0.6935 | 0.6610 | 0.7245 | 0.6330 | 0.5970 | 0.6650 | 0.1835 | 0.1525 | 0.2080 | 0.2940 | 0.2610 | 0.3240 |
| 45 | 0.050 | 2.5 | 0.7485 | 0.7145 | 0.7775 | 0.6840 | 0.6535 | 0.7160 | 0.2245 | 0.1945 | 0.2530 | 0.3345 | 0.3045 | 0.3675 |
| 46 | 0.050 | 3.0 | 0.7655 | 0.7350 | 0.8000 | 0.7050 | 0.6680 | 0.7395 | 0.2740 | 0.2410 | 0.3045 | 0.3895 | 0.3560 | 0.4280 |
| 47 | 0.100 | 1.0 | 0.5980 | 0.5620 | 0.6385 | 0.5055 | 0.4685 | 0.5520 | 0.1450 | 0.1190 | 0.1715 | 0.2165 | 0.1860 | 0.2440 |
| 48 | 0.100 | 1.5 | 0.6935 | 0.6620 | 0.7330 | 0.6150 | 0.5765 | 0.6445 | 0.2130 | 0.1840 | 0.2410 | 0.3160 | 0.2805 | 0.3525 |
| 49 | 0.100 | 2.0 | 0.7390 | 0.7105 | 0.7670 | 0.6655 | 0.6280 | 0.6990 | 0.2820 | 0.2505 | 0.3140 | 0.3860 | 0.3515 | 0.4250 |
| 50 | 0.100 | 2.5 | 0.7775 | 0.7500 | 0.8075 | 0.7100 | 0.6775 | 0.7485 | 0.3205 | 0.2910 | 0.3520 | 0.4060 | 0.3670 | 0.4375 |
| 51 | 0.100 | 3.0 | 0.8205 | 0.7910 | 0.8460 | 0.7470 | 0.7170 | 0.7800 | 0.3855 | 0.3475 | 0.4165 | 0.4840 | 0.4465 | 0.5165 |
| 52 | 0.150 | 1.0 | 0.6240 | 0.5935 | 0.6580 | 0.5135 | 0.4815 | 0.5500 | 0.2035 | 0.1755 | 0.2365 | 0.2640 | 0.2395 | 0.2920 |
| 53 | 0.150 | 1.5 | 0.7020 | 0.6625 | 0.7320 | 0.5940 | 0.5585 | 0.6335 | 0.2730 | 0.2375 | 0.3070 | 0.3395 | 0.3000 | 0.3710 |
| 54 | 0.150 | 2.0 | 0.7670 | 0.7350 | 0.7965 | 0.6755 | 0.6420 | 0.7125 | 0.3460 | 0.3055 | 0.3795 | 0.4205 | 0.3885 | 0.4655 |
| 55 | 0.150 | 2.5 | 0.8035 | 0.7690 | 0.8315 | 0.7275 | 0.6945 | 0.7580 | 0.4070 | 0.3760 | 0.4420 | 0.4785 | 0.4360 | 0.5215 |
| 56 | 0.150 | 3.0 | 0.8255 | 0.7980 | 0.8530 | 0.7615 | 0.7290 | 0.7905 | 0.4595 | 0.4205 | 0.4985 | 0.5085 | 0.4770 | 0.5445 |
| 57 | 0.200 | 1.0 | 0.6310 | 0.6020 | 0.6655 | 0.5095 | 0.4770 | 0.5440 | 0.2455 | 0.2195 | 0.2740 | 0.2695 | 0.2380 | 0.3020 |
| 58 | 0.200 | 1.5 | 0.7390 | 0.7095 | 0.7670 | 0.6315 | 0.6000 | 0.6645 | 0.3240 | 0.2900 | 0.3540 | 0.3555 | 0.3170 | 0.3915 |
| 59 | 0.200 | 2.0 | 0.7855 | 0.7520 | 0.8115 | 0.7000 | 0.6645 | 0.7310 | 0.4115 | 0.3730 | 0.4490 | 0.4355 | 0.4015 | 0.4725 |
| 60 | 0.200 | 2.5 | 0.8070 | 0.7780 | 0.8410 | 0.7165 | 0.6805 | 0.7480 | 0.4500 | 0.4195 | 0.4815 | 0.4820 | 0.4455 | 0.5120 |
| 61 | 0.200 | 3.0 | 0.8450 | 0.8160 | 0.8665 | 0.7625 | 0.7325 | 0.7950 | 0.5225 | 0.4870 | 0.5590 | 0.5555 | 0.5175 | 0.5875 |
| 62 | 0.250 | 1.0 | 0.6615 | 0.6200 | 0.6985 | 0.5235 | 0.4870 | 0.5610 | 0.2770 | 0.2415 | 0.3050 | 0.2915 | 0.2590 | 0.3245 |
| 63 | 0.250 | 1.5 | 0.7265 | 0.6930 | 0.7585 | 0.6110 | 0.5730 | 0.6430 | 0.3495 | 0.3115 | 0.3825 | 0.3790 | 0.3365 | 0.4150 |
| 64 | 0.250 | 2.0 | 0.7945 | 0.7665 | 0.8210 | 0.6970 | 0.6665 | 0.7290 | 0.4390 | 0.4065 | 0.4710 | 0.4465 | 0.4125 | 0.4830 |
| 65 | 0.250 | 2.5 | 0.8285 | 0.8025 | 0.8550 | 0.7340 | 0.6940 | 0.7645 | 0.4985 | 0.4645 | 0.5375 | 0.5050 | 0.4650 | 0.5420 |
| 66 | 0.250 | 3.0 | 0.8715 | 0.8490 | 0.8940 | 0.7845 | 0.7510 | 0.8095 | 0.5490 | 0.5130 | 0.5800 | 0.5645 | 0.5290 | 0.6010 |
| 67 | 0.300 | 1.0 | 0.6735 | 0.6410 | 0.7085 | 0.5290 | 0.4900 | 0.5690 | 0.3220 | 0.2890 | 0.3545 | 0.2885 | 0.2580 | 0.3280 |
| 68 | 0.300 | 1.5 | 0.7425 | 0.7120 | 0.7810 | 0.6055 | 0.5685 | 0.6430 | 0.3910 | 0.3540 | 0.4255 | 0.3835 | 0.3555 | 0.4250 |
| 69 | 0.300 | 2.0 | 0.7945 | 0.7670 | 0.8200 | 0.6865 | 0.6550 | 0.7180 | 0.4870 | 0.4465 | 0.5205 | 0.4465 | 0.4100 | 0.4850 |
| 70 | 0.300 | 2.5 | 0.8280 | 0.8015 | 0.8555 | 0.7390 | 0.7100 | 0.7720 | 0.5385 | 0.5020 | 0.5775 | 0.5045 | 0.4630 | 0.5425 |
| 71 | 0.300 | 3.0 | 0.8455 | 0.8230 | 0.8715 | 0.7590 | 0.7295 | 0.7880 | 0.5720 | 0.5370 | 0.6065 | 0.5330 | 0.4870 | 0.5645 |
| 72 | 0.400 | 1.0 | 0.6665 | 0.6310 | 0.6995 | 0.5155 | 0.4715 | 0.5480 | 0.3520 | 0.3150 | 0.3840 | 0.2680 | 0.2320 | 0.3030 |
| 73 | 0.400 | 1.5 | 0.7640 | 0.7320 | 0.7975 | 0.6270 | 0.5905 | 0.6725 | 0.4605 | 0.4260 | 0.5005 | 0.3815 | 0.3480 | 0.4220 |
| 74 | 0.400 | 2.0 | 0.8035 | 0.7700 | 0.8330 | 0.6795 | 0.6510 | 0.7115 | 0.5565 | 0.5155 | 0.5860 | 0.4540 | 0.4195 | 0.4910 |

| | | | | | | | | | | | | | | |
|---|---|---|---|---|---|---|---|---|---|---|---|---|---|---|
| 75 | 0.400 | 2.5 | 0.8290 | 0.8040 | 0.8520 | 0.7240 | 0.6930 | 0.7560 | 0.5765 | 0.5435 | 0.6110 | 0.5160 | 0.4875 | 0.5545 |
| 76 | 0.400 | 3.0 | 0.8555 | 0.8270 | 0.8800 | 0.7475 | 0.7090 | 0.7750 | 0.6205 | 0.5835 | 0.6595 | 0.5440 | 0.5125 | 0.5845 |
| 77 | 0.500 | 1.0 | 0.6705 | 0.6360 | 0.7040 | 0.5185 | 0.4865 | 0.5480 | 0.3890 | 0.3510 | 0.4175 | 0.2930 | 0.2560 | 0.3290 |
| 78 | 0.500 | 1.5 | 0.7745 | 0.7475 | 0.8045 | 0.6320 | 0.5955 | 0.6640 | 0.5070 | 0.4720 | 0.5470 | 0.3770 | 0.3375 | 0.4095 |
| 79 | 0.500 | 2.0 | 0.8175 | 0.7915 | 0.8435 | 0.6985 | 0.6685 | 0.7365 | 0.5775 | 0.5380 | 0.6160 | 0.4520 | 0.4165 | 0.4905 |
| 80 | 0.500 | 2.5 | 0.8495 | 0.8245 | 0.8720 | 0.7410 | 0.7135 | 0.7695 | 0.6400 | 0.5980 | 0.6745 | 0.5135 | 0.4780 | 0.5475 |
| 81 | 0.500 | 3.0 | 0.8460 | 0.8155 | 0.8745 | 0.7565 | 0.7240 | 0.7930 | 0.6540 | 0.6230 | 0.6865 | 0.5170 | 0.4840 | 0.5555 |
| 82 | 0.600 | 1.0 | 0.6945 | 0.6630 | 0.7285 | 0.5280 | 0.4885 | 0.5675 | 0.4300 | 0.3880 | 0.4630 | 0.2745 | 0.2420 | 0.3050 |
| 83 | 0.600 | 1.5 | 0.7725 | 0.7410 | 0.8030 | 0.6135 | 0.5740 | 0.6525 | 0.5380 | 0.5040 | 0.5730 | 0.3780 | 0.3445 | 0.4080 |
| 84 | 0.600 | 2.0 | 0.8205 | 0.7925 | 0.8440 | 0.6905 | 0.6585 | 0.7255 | 0.6030 | 0.5700 | 0.6450 | 0.4405 | 0.4080 | 0.4740 |
| 85 | 0.600 | 2.5 | 0.8590 | 0.8300 | 0.8860 | 0.7380 | 0.7075 | 0.7670 | 0.6555 | 0.6155 | 0.6855 | 0.5150 | 0.4745 | 0.5550 |
| 86 | 0.600 | 3.0 | 0.8805 | 0.8580 | 0.9050 | 0.7825 | 0.7565 | 0.8145 | 0.7100 | 0.6785 | 0.7445 | 0.5615 | 0.5265 | 0.6010 |
| 87 | 0.700 | 1.0 | 0.7020 | 0.6650 | 0.7340 | 0.5120 | 0.4735 | 0.5515 | 0.4585 | 0.4210 | 0.4990 | 0.2920 | 0.2610 | 0.3245 |
| 88 | 0.700 | 1.5 | 0.7735 | 0.7440 | 0.8050 | 0.6195 | 0.5890 | 0.6510 | 0.5360 | 0.5030 | 0.5790 | 0.3770 | 0.3415 | 0.4130 |
| 89 | 0.700 | 2.0 | 0.8235 | 0.7975 | 0.8535 | 0.6970 | 0.6655 | 0.7375 | 0.6275 | 0.5950 | 0.6605 | 0.4490 | 0.4095 | 0.4855 |
| 90 | 0.700 | 2.5 | 0.8515 | 0.8265 | 0.8750 | 0.7255 | 0.6915 | 0.7575 | 0.6770 | 0.6490 | 0.7115 | 0.5010 | 0.4655 | 0.5305 |
| 91 | 0.700 | 3.0 | 0.8750 | 0.8470 | 0.8990 | 0.7685 | 0.7360 | 0.8010 | 0.7100 | 0.6725 | 0.7395 | 0.5440 | 0.5105 | 0.5840 |
| 92 | 0.800 | 1.0 | 0.7095 | 0.6745 | 0.7440 | 0.5200 | 0.4870 | 0.5540 | 0.4790 | 0.4335 | 0.5115 | 0.2845 | 0.2525 | 0.3180 |
| 93 | 0.800 | 1.5 | 0.7685 | 0.7390 | 0.8010 | 0.6120 | 0.5775 | 0.6440 | 0.5830 | 0.5535 | 0.6155 | 0.3845 | 0.3455 | 0.4295 |
| 94 | 0.800 | 2.0 | 0.8210 | 0.7900 | 0.8480 | 0.6590 | 0.6220 | 0.6925 | 0.6510 | 0.6145 | 0.6815 | 0.4350 | 0.4015 | 0.4670 |
| 95 | 0.800 | 2.5 | 0.8605 | 0.8345 | 0.8910 | 0.7325 | 0.6985 | 0.7640 | 0.7045 | 0.6665 | 0.7410 | 0.5090 | 0.4745 | 0.5420 |
| 96 | 0.800 | 3.0 | 0.8820 | 0.8520 | 0.9065 | 0.7665 | 0.7365 | 0.7940 | 0.7385 | 0.7080 | 0.7755 | 0.5570 | 0.5215 | 0.5880 |
| 97 | 0.900 | 1.0 | 0.7110 | 0.6770 | 0.7420 | 0.5180 | 0.4815 | 0.5510 | 0.4885 | 0.4560 | 0.5235 | 0.3060 | 0.2755 | 0.3375 |
| 98 | 0.900 | 1.5 | 0.7890 | 0.7530 | 0.8160 | 0.6285 | 0.5925 | 0.6660 | 0.5845 | 0.5450 | 0.6165 | 0.3895 | 0.3595 | 0.4305 |
| 99 | 0.900 | 2.0 | 0.8255 | 0.8000 | 0.8555 | 0.6905 | 0.6580 | 0.7300 | 0.6370 | 0.6045 | 0.6760 | 0.4430 | 0.4100 | 0.4750 |
| 100 | 0.900 | 2.5 | 0.8560 | 0.8320 | 0.8795 | 0.7305 | 0.6980 | 0.7650 | 0.7130 | 0.6855 | 0.7510 | 0.5070 | 0.4720 | 0.5455 |
| 101 | 0.900 | 3.0 | 0.8810 | 0.8545 | 0.9085 | 0.7690 | 0.7350 | 0.7955 | 0.7455 | 0.7170 | 0.7915 | 0.5440 | 0.5085 | 0.5855 |
| 102 | 1.000 | 1.0 | 0.7130 | 0.6790 | 0.7495 | 0.5170 | 0.4715 | 0.5525 | 0.4980 | 0.4630 | 0.5355 | 0.3060 | 0.2750 | 0.3400 |
| 103 | 1.000 | 1.5 | 0.7740 | 0.7480 | 0.8025 | 0.6100 | 0.5770 | 0.6415 | 0.5585 | 0.5195 | 0.5935 | 0.3605 | 0.3250 | 0.3945 |
| 104 | 1.000 | 2.0 | 0.8395 | 0.8110 | 0.8655 | 0.6955 | 0.6555 | 0.7280 | 0.6575 | 0.6245 | 0.6980 | 0.4575 | 0.4205 | 0.4940 |
| 105 | 1.000 | 2.5 | 0.8545 | 0.8295 | 0.8785 | 0.7085 | 0.6755 | 0.7425 | 0.7040 | 0.6690 | 0.7360 | 0.5065 | 0.4675 | 0.5395 |
| 106 | 1.000 | 3.0 | 0.8835 | 0.8610 | 0.9060 | 0.7740 | 0.7460 | 0.8045 | 0.7450 | 0.7105 | 0.7740 | 0.5365 | 0.5055 | 0.5695 |

**Average number of SIC according to transmission probability and number of contacts that nurses had with patients: analysis by population (patients, nurses, and physicians)**

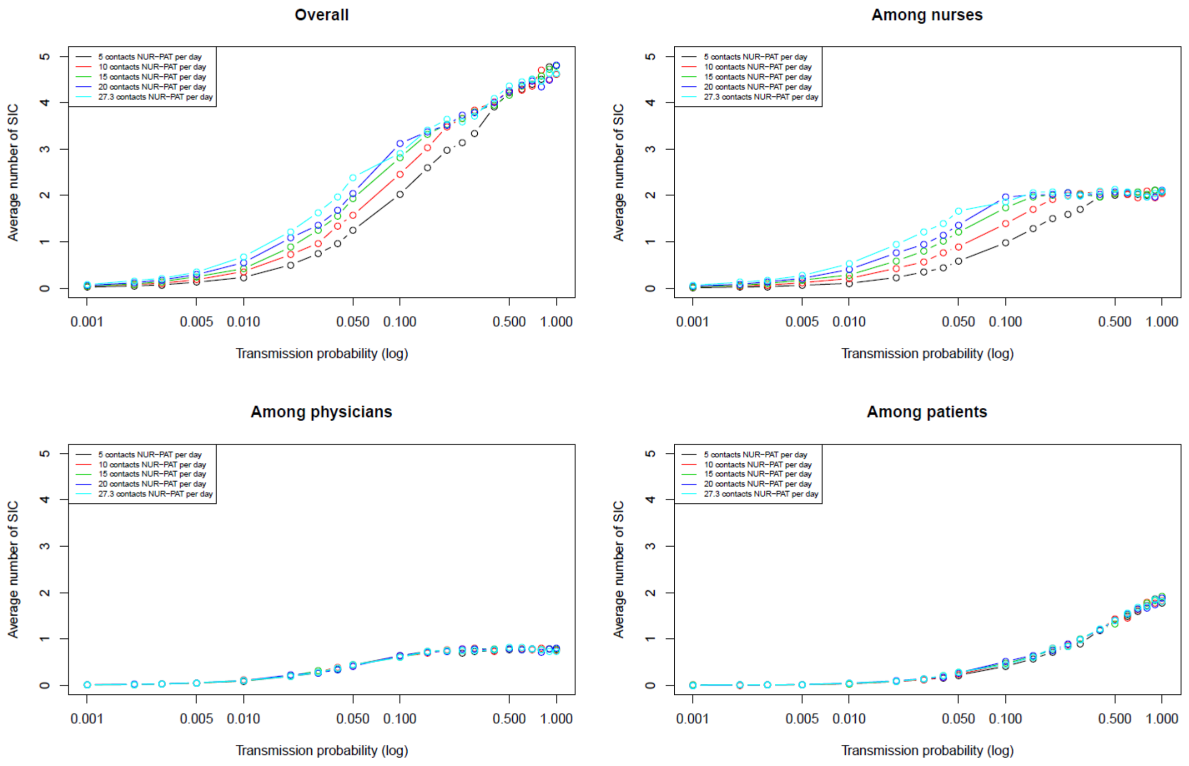


| | Transmission probability | Daily contacts of NUR with PAT | Overall SIC | NUR SIC | PHY SIC | PAT SIC |
|---|---|---|---|---|---|---|
| 1 | 0.001 | 5.0 | 0.0250 | 0.0100 | 0.0115 | 0.0035 |
| 2 | 0.002 | 5.0 | 0.0470 | 0.0245 | 0.0170 | 0.0055 |
| 3 | 0.003 | 5.0 | 0.0725 | 0.0340 | 0.0270 | 0.0115 |
| 4 | 0.005 | 5.0 | 0.1285 | 0.0625 | 0.0515 | 0.0145 |
| 5 | 0.010 | 5.0 | 0.2330 | 0.1050 | 0.0935 | 0.0345 |
| 6 | 0.020 | 5.0 | 0.5035 | 0.2305 | 0.1935 | 0.0795 |
| 7 | 0.030 | 5.0 | 0.7450 | 0.3495 | 0.2685 | 0.1270 |
| 8 | 0.040 | 5.0 | 0.9550 | 0.4365 | 0.3595 | 0.1590 |
| 9 | 0.050 | 5.0 | 1.2450 | 0.5855 | 0.4405 | 0.2190 |
| 10 | 0.100 | 5.0 | 2.0260 | 0.9790 | 0.6365 | 0.4105 |
| 11 | 0.150 | 5.0 | 2.5925 | 1.2820 | 0.7375 | 0.5730 |
| 12 | 0.200 | 5.0 | 2.9715 | 1.5115 | 0.7420 | 0.7180 |
| 13 | 0.250 | 5.0 | 3.1385 | 1.5985 | 0.6925 | 0.8475 |
| 14 | 0.300 | 5.0 | 3.3340 | 1.7015 | 0.7290 | 0.9035 |
| 15 | 0.400 | 5.0 | 3.9090 | 1.9725 | 0.7490 | 1.1875 |
| 16 | 0.500 | 5.0 | 4.2115 | 2.0150 | 0.7760 | 1.4205 |
| 17 | 0.600 | 5.0 | 4.2950 | 2.0315 | 0.7815 | 1.4820 |
| 18 | 0.700 | 5.0 | 4.4055 | 2.0275 | 0.7820 | 1.5960 |
| 19 | 0.800 | 5.0 | 4.5060 | 2.0140 | 0.7615 | 1.7305 |
| 20 | 0.900 | 5.0 | 4.7760 | 2.1120 | 0.7895 | 1.8745 |
| 21 | 1.000 | 5.0 | 4.6230 | 2.0800 | 0.7660 | 1.7770 |
| 22 | 0.001 | 10.0 | 0.0510 | 0.0300 | 0.0145 | 0.0065 |
| 23 | 0.002 | 10.0 | 0.0795 | 0.0550 | 0.0195 | 0.0050 |
| 24 | 0.003 | 10.0 | 0.1070 | 0.0660 | 0.0315 | 0.0095 |
| 25 | 0.005 | 10.0 | 0.1815 | 0.1190 | 0.0455 | 0.0170 |
| 26 | 0.010 | 10.0 | 0.3605 | 0.2080 | 0.1145 | 0.0380 |
| 27 | 0.020 | 10.0 | 0.7230 | 0.4310 | 0.2065 | 0.0855 |
| 28 | 0.030 | 10.0 | 0.9710 | 0.5670 | 0.2830 | 0.1210 |
| 29 | 0.040 | 10.0 | 1.3475 | 0.7685 | 0.3995 | 0.1795 |
| 30 | 0.050 | 10.0 | 1.5695 | 0.8955 | 0.4275 | 0.2465 |
| 31 | 0.100 | 10.0 | 2.4645 | 1.3890 | 0.6265 | 0.4490 |
| 32 | 0.150 | 10.0 | 3.0230 | 1.7095 | 0.6895 | 0.6240 |
| 33 | 0.200 | 10.0 | 3.4880 | 1.9240 | 0.7650 | 0.7990 |
| 34 | 0.250 | 10.0 | 3.6550 | 1.9985 | 0.7790 | 0.8775 |
| 35 | 0.300 | 10.0 | 3.8440 | 2.0500 | 0.8010 | 0.9930 |
| 36 | 0.400 | 10.0 | 4.0125 | 2.0735 | 0.7405 | 1.1985 |
| 37 | 0.500 | 10.0 | 4.2580 | 2.0390 | 0.7830 | 1.4360 |

| | | | | | | |
|---|---|---|---|---|---|---|
| 38 | 0.600 | 10.0 | 4.2645 | 2.0290 | 0.7825 | 1.4530 |
| 39 | 0.700 | 10.0 | 4.3685 | 1.9575 | 0.7700 | 1.6410 |
| 40 | 0.800 | 10.0 | 4.7035 | 2.1050 | 0.8075 | 1.7910 |
| 41 | 0.900 | 10.0 | 4.5115 | 1.9515 | 0.7885 | 1.7715 |
| 42 | 1.000 | 10.0 | 4.6145 | 2.0455 | 0.7440 | 1.8250 |
| 43 | 0.001 | 15.0 | 0.0520 | 0.0300 | 0.0140 | 0.0080 |
| 44 | 0.002 | 15.0 | 0.0880 | 0.0620 | 0.0180 | 0.0080 |
| 45 | 0.003 | 15.0 | 0.1430 | 0.1020 | 0.0320 | 0.0090 |
| 46 | 0.005 | 15.0 | 0.2465 | 0.1745 | 0.0520 | 0.0200 |
| 47 | 0.010 | 15.0 | 0.4215 | 0.2845 | 0.0965 | 0.0405 |
| 48 | 0.020 | 15.0 | 0.8845 | 0.5875 | 0.2060 | 0.0910 |
| 49 | 0.030 | 15.0 | 1.2545 | 0.8015 | 0.3135 | 0.1395 |
| 50 | 0.040 | 15.0 | 1.5575 | 1.0120 | 0.3375 | 0.2080 |
| 51 | 0.050 | 15.0 | 1.9300 | 1.2070 | 0.4415 | 0.2815 |
| 52 | 0.100 | 15.0 | 2.8155 | 1.7350 | 0.6050 | 0.4755 |
| 53 | 0.150 | 15.0 | 3.3215 | 1.9760 | 0.7210 | 0.6245 |
| 54 | 0.200 | 15.0 | 3.5330 | 2.0095 | 0.7595 | 0.7640 |
| 55 | 0.250 | 15.0 | 3.6560 | 2.0620 | 0.7510 | 0.8430 |
| 56 | 0.300 | 15.0 | 3.8120 | 2.0225 | 0.7805 | 1.0090 |
| 57 | 0.400 | 15.0 | 3.9540 | 1.9700 | 0.7815 | 1.2025 |
| 58 | 0.500 | 15.0 | 4.1570 | 2.0395 | 0.7860 | 1.3315 |
| 59 | 0.600 | 15.0 | 4.3645 | 2.0670 | 0.7700 | 1.5275 |
| 60 | 0.700 | 15.0 | 4.5055 | 2.0695 | 0.7845 | 1.6515 |
| 61 | 0.800 | 15.0 | 4.5690 | 2.0230 | 0.7690 | 1.7770 |
| 62 | 0.900 | 15.0 | 4.7300 | 2.1175 | 0.7900 | 1.8225 |
| 63 | 1.000 | 15.0 | 4.7945 | 2.0880 | 0.7840 | 1.9225 |
| 64 | 0.001 | 20.0 | 0.0585 | 0.0425 | 0.0140 | 0.0020 |
| 65 | 0.002 | 20.0 | 0.1160 | 0.0845 | 0.0255 | 0.0060 |
| 66 | 0.003 | 20.0 | 0.1780 | 0.1380 | 0.0325 | 0.0075 |
| 67 | 0.005 | 20.0 | 0.2920 | 0.2120 | 0.0580 | 0.0220 |
| 68 | 0.010 | 20.0 | 0.5525 | 0.4035 | 0.1015 | 0.0475 |
| 69 | 0.020 | 20.0 | 1.0845 | 0.7585 | 0.2265 | 0.0995 |
| 70 | 0.030 | 20.0 | 1.3610 | 0.9450 | 0.2700 | 0.1460 |
| 71 | 0.040 | 20.0 | 1.6740 | 1.1520 | 0.3370 | 0.1850 |
| 72 | 0.050 | 20.0 | 2.0500 | 1.3675 | 0.4120 | 0.2705 |
| 73 | 0.100 | 20.0 | 3.1200 | 1.9645 | 0.6435 | 0.5120 |
| 74 | 0.150 | 20.0 | 3.3770 | 2.0090 | 0.7165 | 0.6515 |
| 75 | 0.200 | 20.0 | 3.5140 | 2.0175 | 0.7360 | 0.7605 |
| 76 | 0.250 | 20.0 | 3.7320 | 2.0590 | 0.7790 | 0.8940 |
| 77 | 0.300 | 20.0 | 3.7825 | 2.0080 | 0.7830 | 0.9915 |
| 78 | 0.400 | 20.0 | 4.0050 | 2.0255 | 0.7755 | 1.2040 |
| 79 | 0.500 | 20.0 | 4.2495 | 2.0735 | 0.7840 | 1.3920 |
| 80 | 0.600 | 20.0 | 4.3790 | 2.0670 | 0.7640 | 1.5480 |
| 81 | 0.700 | 20.0 | 4.4785 | 2.0385 | 0.7805 | 1.6595 |
| 82 | 0.800 | 20.0 | 4.3530 | 1.9705 | 0.7190 | 1.6635 |
| 83 | 0.900 | 20.0 | 4.4955 | 1.9745 | 0.7840 | 1.7370 |
| 84 | 1.000 | 20.0 | 4.8060 | 2.1205 | 0.8040 | 1.8815 |
| 85 | 0.001 | 27.3 | 0.0775 | 0.0620 | 0.0115 | 0.0040 |
| 86 | 0.002 | 27.3 | 0.1630 | 0.1285 | 0.0230 | 0.0115 |
| 87 | 0.003 | 27.3 | 0.2110 | 0.1720 | 0.0280 | 0.0110 |
| 88 | 0.005 | 27.3 | 0.3445 | 0.2715 | 0.0555 | 0.0175 |
| 89 | 0.010 | 27.3 | 0.6810 | 0.5270 | 0.1075 | 0.0465 |
| 90 | 0.020 | 27.3 | 1.2240 | 0.9405 | 0.1925 | 0.0910 |
| 91 | 0.030 | 27.3 | 1.6370 | 1.2155 | 0.2815 | 0.1400 |
| 92 | 0.040 | 27.3 | 1.9705 | 1.3925 | 0.3685 | 0.2095 |
| 93 | 0.050 | 27.3 | 2.3900 | 1.6670 | 0.4420 | 0.2810 |
| 94 | 0.100 | 27.3 | 2.9110 | 1.8585 | 0.6055 | 0.4470 |
| 95 | 0.150 | 27.3 | 3.4130 | 2.0650 | 0.7340 | 0.6140 |
| 96 | 0.200 | 27.3 | 3.6460 | 2.0780 | 0.7570 | 0.8110 |
| 97 | 0.250 | 27.3 | 3.5870 | 1.9935 | 0.7485 | 0.8450 |
| 98 | 0.300 | 27.3 | 3.7180 | 1.9875 | 0.7435 | 0.9870 |
| 99 | 0.400 | 27.3 | 4.0895 | 2.1040 | 0.7615 | 1.2240 |
| 100 | 0.500 | 27.3 | 4.3600 | 2.1360 | 0.8220 | 1.4020 |
| 101 | 0.600 | 27.3 | 4.4525 | 2.0750 | 0.8205 | 1.5570 |
| 102 | 0.700 | 27.3 | 4.5025 | 2.0345 | 0.7880 | 1.6800 |
| 103 | 0.800 | 27.3 | 4.4695 | 1.9775 | 0.7785 | 1.7135 |
| 104 | 0.900 | 27.3 | 4.6345 | 2.0360 | 0.7415 | 1.8570 |
| 105 | 1.000 | 27.3 | 4.6405 | 2.0990 | 0.7325 | 1.8090 |

**Average number of SIC according to transmission probability and duration of the dry phase of EVD of the index case: analysis by population (patients, nurses, and physicians)**

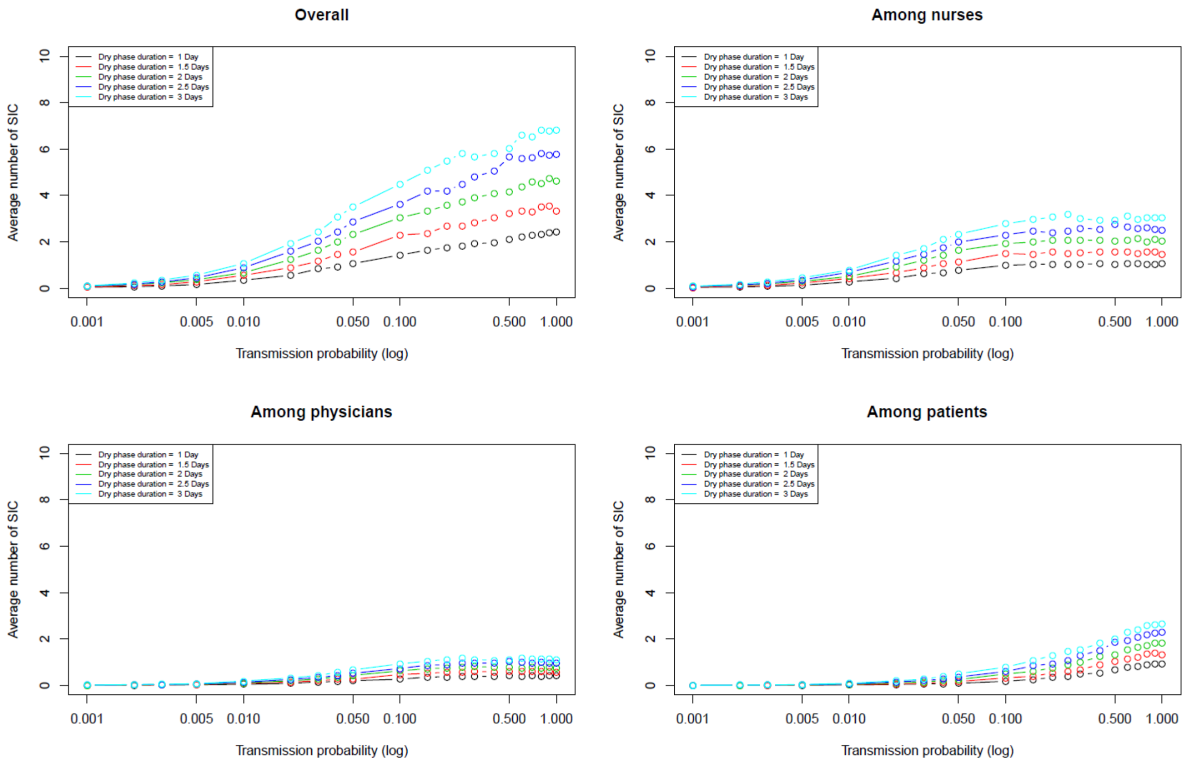


| | Transmission probability | Dry phase duration (days) | Overall SIC | NUR SIC | PHY SIC | PAT SIC |
|---|---|---|---|---|---|---|
| 1 | 0.001 | 1.0 | 0.0430 | 0.0345 | 0.0060 | 0.0025 |
| 2 | 0.002 | 1.0 | 0.0635 | 0.0525 | 0.0085 | 0.0025 |
| 3 | 0.003 | 1.0 | 0.0985 | 0.0800 | 0.0155 | 0.0030 |
| 4 | 0.005 | 1.0 | 0.1575 | 0.1250 | 0.0250 | 0.0075 |
| 5 | 0.010 | 1.0 | 0.3460 | 0.2795 | 0.0480 | 0.0185 |
| 6 | 0.020 | 1.0 | 0.5570 | 0.4305 | 0.0850 | 0.0415 |
| 7 | 0.030 | 1.0 | 0.8330 | 0.6175 | 0.1510 | 0.0645 |
| 8 | 0.040 | 1.0 | 0.9090 | 0.6765 | 0.1620 | 0.0705 |
| 9 | 0.050 | 1.0 | 1.0625 | 0.7725 | 0.2010 | 0.0890 |
| 10 | 0.100 | 1.0 | 1.4305 | 0.9865 | 0.2670 | 0.1770 |
| 11 | 0.150 | 1.0 | 1.6435 | 1.0255 | 0.3580 | 0.2600 |
| 12 | 0.200 | 1.0 | 1.7485 | 1.0435 | 0.3690 | 0.3360 |
| 13 | 0.250 | 1.0 | 1.8240 | 1.0410 | 0.3835 | 0.3995 |
| 14 | 0.300 | 1.0 | 1.9215 | 1.0450 | 0.3980 | 0.4785 |
| 15 | 0.400 | 1.0 | 1.9595 | 1.0485 | 0.3710 | 0.5400 |
| 16 | 0.500 | 1.0 | 2.1180 | 1.0360 | 0.4075 | 0.6745 |
| 17 | 0.600 | 1.0 | 2.2025 | 1.0510 | 0.3785 | 0.7730 |
| 18 | 0.700 | 1.0 | 2.2860 | 1.0575 | 0.4065 | 0.8220 |
| 19 | 0.800 | 1.0 | 2.3130 | 1.0300 | 0.4015 | 0.8815 |
| 20 | 0.900 | 1.0 | 2.3865 | 1.0370 | 0.4215 | 0.9280 |
| 21 | 1.000 | 1.0 | 2.4190 | 1.0665 | 0.4155 | 0.9370 |
| 22 | 0.001 | 1.5 | 0.0480 | 0.0380 | 0.0060 | 0.0040 |
| 23 | 0.002 | 1.5 | 0.1170 | 0.0970 | 0.0165 | 0.0035 |
| 24 | 0.003 | 1.5 | 0.1470 | 0.1205 | 0.0200 | 0.0065 |
| 25 | 0.005 | 1.5 | 0.2820 | 0.2195 | 0.0465 | 0.0160 |
| 26 | 0.010 | 1.5 | 0.5530 | 0.4245 | 0.0925 | 0.0360 |
| 27 | 0.020 | 1.5 | 0.8935 | 0.6780 | 0.1435 | 0.0720 |
| 28 | 0.030 | 1.5 | 1.1555 | 0.8705 | 0.1960 | 0.0890 |
| 29 | 0.040 | 1.5 | 1.4700 | 1.0700 | 0.2665 | 0.1335 |
| 30 | 0.050 | 1.5 | 1.5500 | 1.1195 | 0.2640 | 0.1665 |
| 31 | 0.100 | 1.5 | 2.2945 | 1.5010 | 0.4715 | 0.3220 |
| 32 | 0.150 | 1.5 | 2.3655 | 1.4560 | 0.5155 | 0.3940 |
| 33 | 0.200 | 1.5 | 2.6905 | 1.5785 | 0.5755 | 0.5365 |
| 34 | 0.250 | 1.5 | 2.6845 | 1.4940 | 0.5805 | 0.6100 |
| 35 | 0.300 | 1.5 | 2.8100 | 1.5270 | 0.5960 | 0.6870 |
| 36 | 0.400 | 1.5 | 3.0330 | 1.5595 | 0.5840 | 0.8895 |
| 37 | 0.500 | 1.5 | 3.2365 | 1.5800 | 0.6065 | 1.0500 |

| | | | | | | |
|---|---|---|---|---|---|---|
| 38 | 0.600 | 1.5 | 3.3465 | 1.5815 | 0.6100 | 1.1550 |
| 39 | 0.700 | 1.5 | 3.2945 | 1.4960 | 0.5945 | 1.2040 |
| 40 | 0.800 | 1.5 | 3.5085 | 1.5520 | 0.6110 | 1.3455 |
| 41 | 0.900 | 1.5 | 3.5485 | 1.5620 | 0.6030 | 1.3835 |
| 42 | 1.000 | 1.5 | 3.3170 | 1.4555 | 0.5550 | 1.3065 |
| 43 | 0.001 | 2.0 | 0.0880 | 0.0750 | 0.0105 | 0.0025 |
| 44 | 0.002 | 2.0 | 0.1550 | 0.1215 | 0.0235 | 0.0100 |
| 45 | 0.003 | 2.0 | 0.2350 | 0.1845 | 0.0380 | 0.0125 |
| 46 | 0.005 | 2.0 | 0.3635 | 0.2930 | 0.0480 | 0.0225 |
| 47 | 0.010 | 2.0 | 0.6715 | 0.5090 | 0.1165 | 0.0460 |
| 48 | 0.020 | 2.0 | 1.2320 | 0.9250 | 0.1950 | 0.1120 |
| 49 | 0.030 | 2.0 | 1.6295 | 1.2090 | 0.2780 | 0.1425 |
| 50 | 0.040 | 2.0 | 2.0135 | 1.4360 | 0.3645 | 0.2130 |
| 51 | 0.050 | 2.0 | 2.3110 | 1.6255 | 0.4330 | 0.2525 |
| 52 | 0.100 | 2.0 | 3.0380 | 1.9225 | 0.6245 | 0.4910 |
| 53 | 0.150 | 2.0 | 3.3190 | 1.9900 | 0.7250 | 0.6040 |
| 54 | 0.200 | 2.0 | 3.5820 | 2.0730 | 0.7485 | 0.7605 |
| 55 | 0.250 | 2.0 | 3.7195 | 2.0625 | 0.7795 | 0.8775 |
| 56 | 0.300 | 2.0 | 3.8925 | 2.0815 | 0.8050 | 1.0060 |
| 57 | 0.400 | 2.0 | 4.0870 | 2.0615 | 0.7905 | 1.2350 |
| 58 | 0.500 | 2.0 | 4.1510 | 2.0375 | 0.7795 | 1.3340 |
| 59 | 0.600 | 2.0 | 4.3645 | 2.0715 | 0.7540 | 1.5390 |
| 60 | 0.700 | 2.0 | 4.5845 | 2.1385 | 0.7925 | 1.6535 |
| 61 | 0.800 | 2.0 | 4.5050 | 2.0100 | 0.7600 | 1.7350 |
| 62 | 0.900 | 2.0 | 4.7195 | 2.1100 | 0.7815 | 1.8280 |
| 63 | 1.000 | 2.0 | 4.6405 | 2.0420 | 0.7595 | 1.8390 |
| 64 | 0.001 | 2.5 | 0.0935 | 0.0725 | 0.0150 | 0.0060 |
| 65 | 0.002 | 2.5 | 0.1765 | 0.1370 | 0.0255 | 0.0140 |
| 66 | 0.003 | 2.5 | 0.2725 | 0.2165 | 0.0410 | 0.0150 |
| 67 | 0.005 | 2.5 | 0.4470 | 0.3555 | 0.0590 | 0.0325 |
| 68 | 0.010 | 2.5 | 0.8840 | 0.6875 | 0.1355 | 0.0610 |
| 69 | 0.020 | 2.5 | 1.5860 | 1.1760 | 0.2585 | 0.1515 |
| 70 | 0.030 | 2.5 | 2.0245 | 1.4760 | 0.3365 | 0.2120 |
| 71 | 0.040 | 2.5 | 2.4370 | 1.7420 | 0.4285 | 0.2665 |
| 72 | 0.050 | 2.5 | 2.8680 | 1.9845 | 0.5215 | 0.3620 |
| 73 | 0.100 | 2.5 | 3.6325 | 2.3040 | 0.7295 | 0.5990 |
| 74 | 0.150 | 2.5 | 4.1765 | 2.4620 | 0.8720 | 0.8425 |
| 75 | 0.200 | 2.5 | 4.2035 | 2.3805 | 0.8815 | 0.9415 |
| 76 | 0.250 | 2.5 | 4.4955 | 2.4690 | 0.9465 | 1.0800 |
| 77 | 0.300 | 2.5 | 4.8175 | 2.5840 | 0.9535 | 1.2800 |
| 78 | 0.400 | 2.5 | 5.0390 | 2.5535 | 0.9665 | 1.5190 |
| 79 | 0.500 | 2.5 | 5.6560 | 2.7580 | 1.0455 | 1.8525 |
| 80 | 0.600 | 2.5 | 5.6005 | 2.6585 | 1.0170 | 1.9250 |
| 81 | 0.700 | 2.5 | 5.6315 | 2.5820 | 0.9635 | 2.0860 |
| 82 | 0.800 | 2.5 | 5.8145 | 2.6190 | 1.0050 | 2.1905 |
| 83 | 0.900 | 2.5 | 5.7310 | 2.5365 | 0.9485 | 2.2460 |
| 84 | 1.000 | 2.5 | 5.7930 | 2.5140 | 0.9810 | 2.2980 |
| 85 | 0.001 | 3.0 | 0.1035 | 0.0840 | 0.0130 | 0.0065 |
| 86 | 0.002 | 3.0 | 0.2230 | 0.1750 | 0.0370 | 0.0110 |
| 87 | 0.003 | 3.0 | 0.3520 | 0.2830 | 0.0490 | 0.0200 |
| 88 | 0.005 | 3.0 | 0.5615 | 0.4490 | 0.0740 | 0.0385 |
| 89 | 0.010 | 3.0 | 1.0670 | 0.7860 | 0.1895 | 0.0915 |
| 90 | 0.020 | 3.0 | 1.9290 | 1.4110 | 0.3185 | 0.1995 |
| 91 | 0.030 | 3.0 | 2.4170 | 1.7270 | 0.4255 | 0.2645 |
| 92 | 0.040 | 3.0 | 3.0755 | 2.1120 | 0.5690 | 0.3945 |
| 93 | 0.050 | 3.0 | 3.4985 | 2.3285 | 0.6670 | 0.5030 |
| 94 | 0.100 | 3.0 | 4.4880 | 2.7755 | 0.9280 | 0.7845 |
| 95 | 0.150 | 3.0 | 5.0745 | 2.9560 | 1.0510 | 1.0675 |
| 96 | 0.200 | 3.0 | 5.4845 | 3.0680 | 1.1180 | 1.2985 |
| 97 | 0.250 | 3.0 | 5.8280 | 3.1785 | 1.1840 | 1.4655 |
| 98 | 0.300 | 3.0 | 5.6605 | 3.0075 | 1.1055 | 1.5475 |
| 99 | 0.400 | 3.0 | 5.8200 | 2.9200 | 1.0890 | 1.8110 |
| 100 | 0.500 | 3.0 | 6.0355 | 2.9355 | 1.0950 | 2.0050 |
| 101 | 0.600 | 3.0 | 6.6185 | 3.1235 | 1.1860 | 2.3090 |
| 102 | 0.700 | 3.0 | 6.5260 | 2.9715 | 1.1370 | 2.4175 |
| 103 | 0.800 | 3.0 | 6.8025 | 3.0475 | 1.1560 | 2.5990 |
| 104 | 0.900 | 3.0 | 6.7830 | 3.0270 | 1.1520 | 2.6040 |
| 105 | 1.000 | 3.0 | 6.8245 | 3.0530 | 1.1055 | 2.6660 |

## Sensitivity analysis of the emergence probability according to the incubation period duration

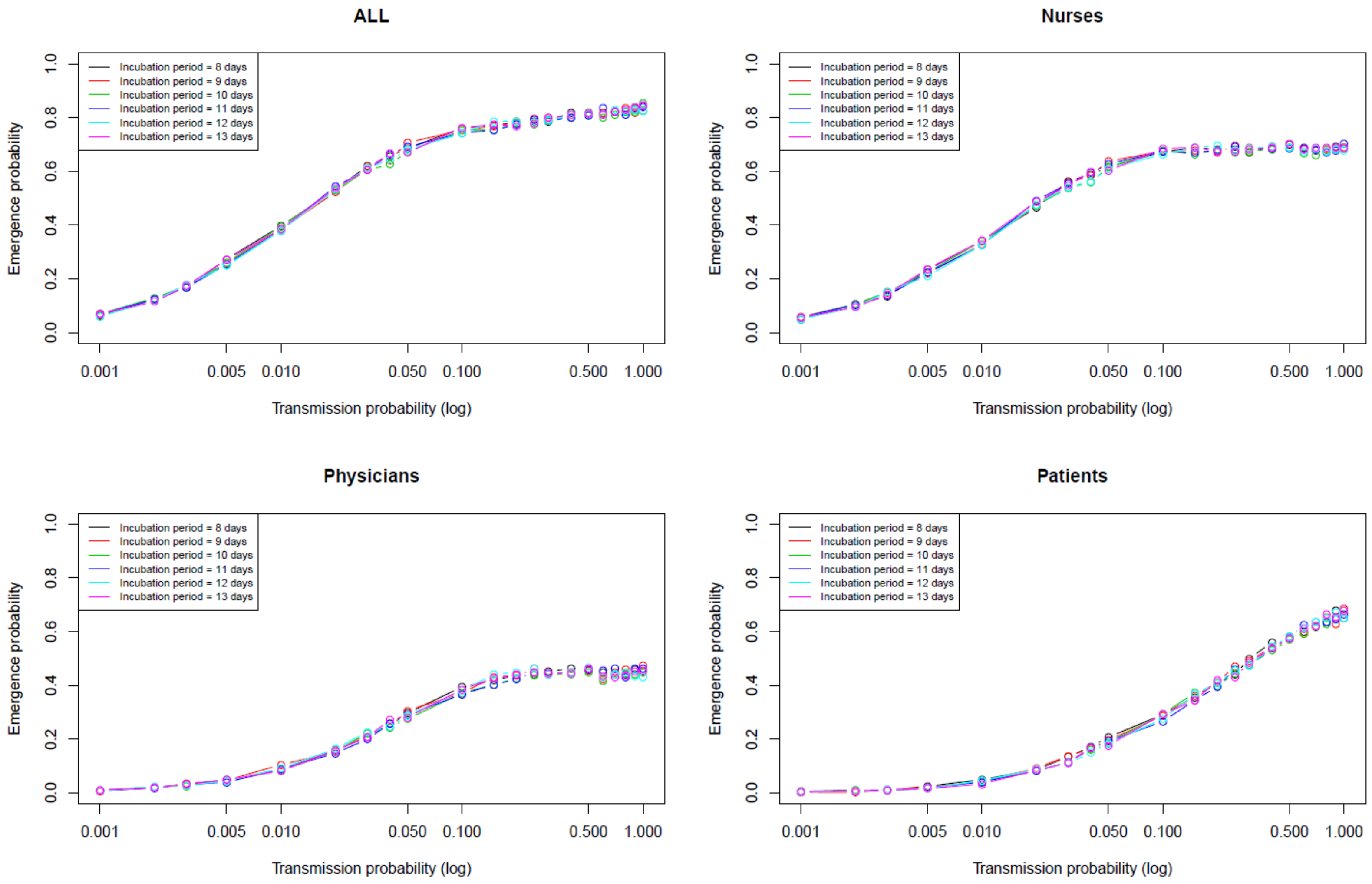

Supplement: Supplementary Information [file srep36301-s1.pdf]
